# Supplementary material for: Accurate predictions of protein mutational effects accelerated with a hybrid-topology free energy protocol
Source: Commun Chem. 2025 Nov 20;8:362. doi: 10.1038/s42004-025-01771-0 (PMC12634679; doi:10.1038/s42004-025-01771-0)
Supplement: Supplementary file 2 — Supplementary Material [file 42004_2025_1771_MOESM2_ESM.pdf]

Accurate Predictions of Protein Mutational Effects Accelerated with a Hybrid-Topology Free Energy Protocol

Lucien Koenekoop[1], Nadine van de Brug[1,§],
Willem Jespers[2,3], Johan Åqvist[1], Hugo Gutiérrez-de-Terán[1,3,4,*]

[1]*Department of Cell & Molecular Biology, Uppsala University, Biomedical Center, SE-75124 Uppsala, Sweden*

[2]*Medicinal Chemistry, Photopharmacology and Imaging, Groningen Research Institute of Pharmacy, NL-9713 AV Groningen, The Netherlands*

[3]*MODSIM Pharma AI B.V., Taxushaag 9, NL-2216 AB Voorhout, The Netherlands*

[4]*Nanomaterials and Nanotechnology Research Center (CINN), CSIC-University of Oviedo-Principado de Asturias, and Health Research Institute of Asturias (ISPA), Av. del Hospital Universitario, s/n, ES-33011 Oviedo, Asturias, Spain.*

[§] Present address: Department of Computer Science, Faculty of Science and Network Institute, Vrije Universiteit of Amsterdam, Amsterdam (NL)
[*]Corresponding author
e-mail: h.g.teran@cinn.es; hugo.gutierrez@icm.uu.se

SUPPLEMENTARY MATERIALS

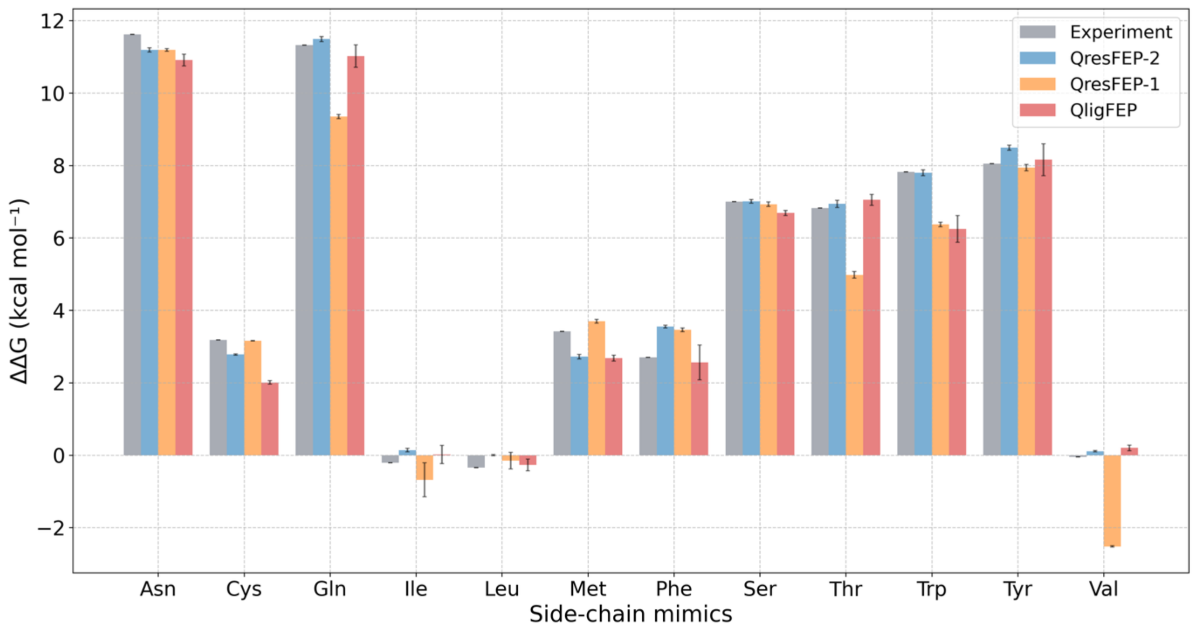

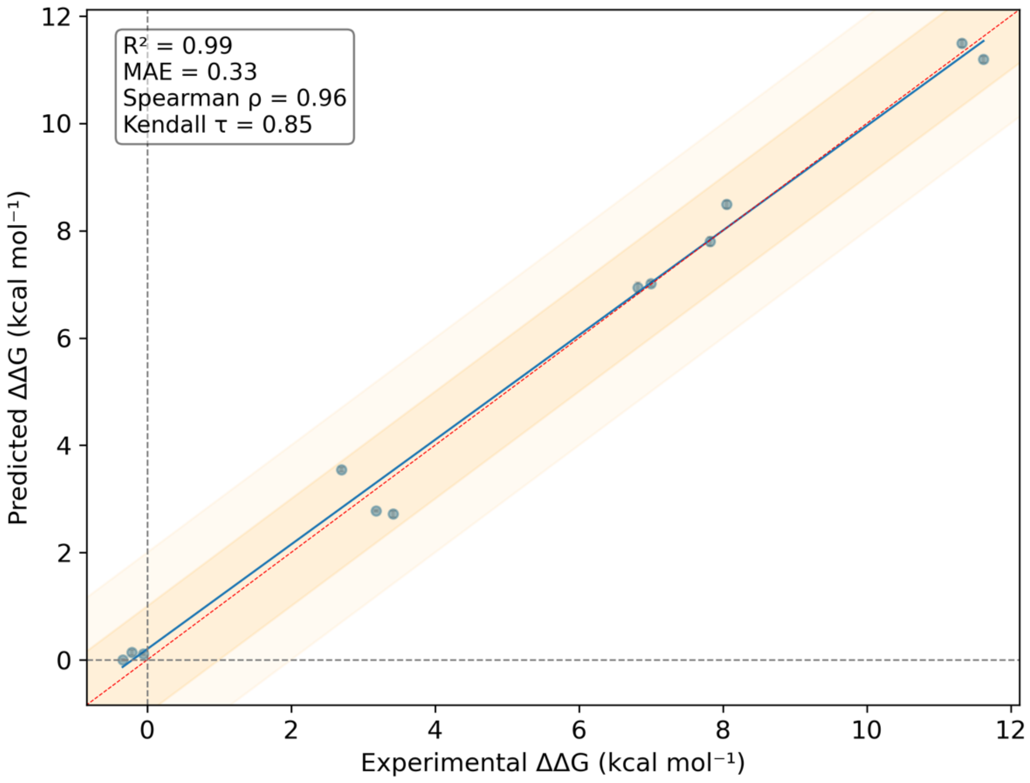

Supplementary Figure 1. A) Solvation free energies ($\Delta\Delta G$) of amino acid side-chain mimics (X) relative to Methane (Me) for hybrid-topology QresFEP-2 (blue), single-topology QresFEP-1 (orange), and dual-topology QligFEP (red), as compared to experiment (grey). Error bars represent standard error of the mean (SEM) calculated from 10 independent simulations. **B)** Correlation plot for experimental (x-axis) vs QresFEP-2 predicted solvation free energies (corresponding to values in blue bars on panel A)

Supplementary Table 1. Performance of this and other methods in the literature on the calculation of hydration free energies of amino acid side chains (X) relative to Methane (Me).

Group	Force field	Subset	*n*	MAE
QligFEP	OPLS-AA/M	neutral	12[a]	0.95
QresFEP-1	OPLS-AA/M	neutral	12[a]	0.85
	OPLS-AA/M	all	18	1.19
	OPLS-AA/M	all (ionized)	23	1.50
QresFEP-2	OPLS-AA/M	neutral	12[a]	0.33
Sandler	OPLS-AA	neutral	14	0.69
Pande	AMBER(ff94)	neutral	15	1.35
	CHARMM22	neutral	15	1.31
	OPLS-AA	neutral	15	0.85
Tieleman	OPLS-AA	all	18	1.71
	Gromos96	all	18	2.41
Mobley	AMBER(GAFF)	all	16	0.99
Van Gunsteren	Gromos43a2	neutral	15	2.08
	Gromos45a3	neutral	15	2.01
	Gromos53a3	neutral	15	1.89
	Gromos53a6	neutral	15	0.19
Pardo	OPLS-AA	all	17	0.93
	AMBER99	all	17	0.67
	AMBER03	all	17	1.48

[a] from the 20 natural amino acids, ionizable side chains (including His) were excluded, together with Pro (side chain embedded on the main chain), Gly (no side chain) and Ala (the reference side chain for pairwise FEP simulations).

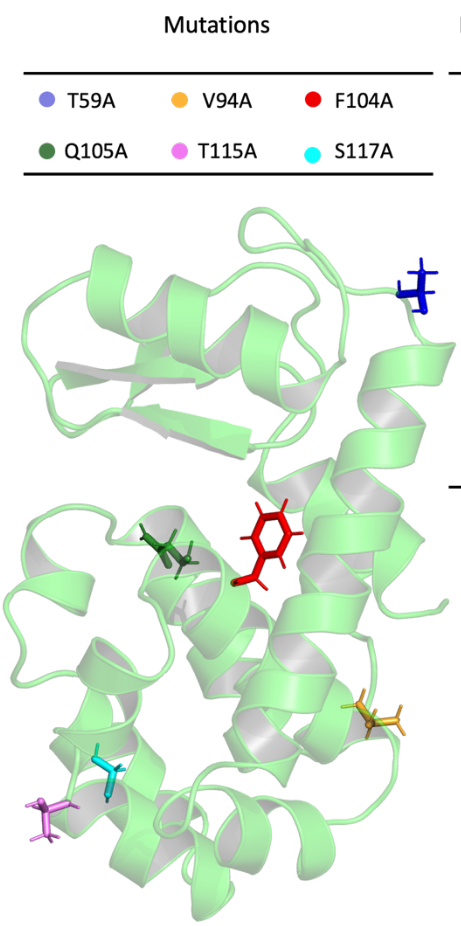

Protocol	t/λ (ps)	λ	Time (ns)	Scheme	MAE (kcal mol^{-1})	SEM (kcal mol^{-1})
A	10	20	4.0	linear	1.80	0.40
B	10	20	4.0	sigmoidal	1.96	0.49
C	10	50	10	linear	1.60	0.39
D	10	50	10	sigmoidal	1.33	0.46
E	20	20	8.0	linear	1.74	0.41
F	20	20	8.0	sigmoidal	1.54	0.43
G	20	50	20	linear	1.51	0.33
H	20	50	20	sigmoidal	1.07	0.37
I	10	100	20	sigmoidal	1.00	0.40
J	20	100	40	sigmoidal	1.09	0.44

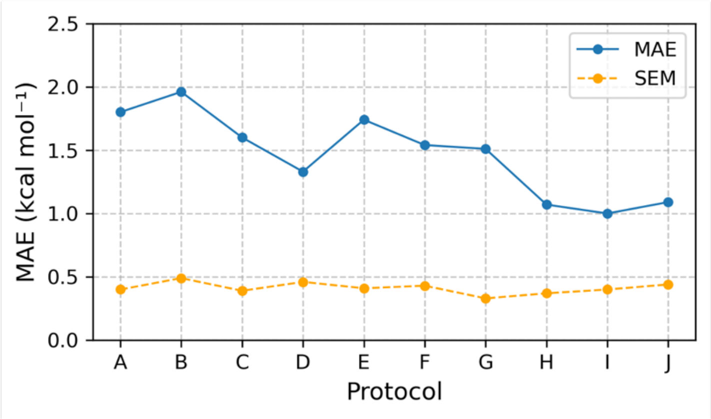

Supplementary Figure 2. Parameters conditioning of the FEP protocol for a subset of six mutations for T4 lysozyme (T4L) transformations representing diverse sizes and chemical properties of the wild-type residues. t/λ = time steps per window, λ = number of windows per FEP stage, Time = total sampling time for a full transformation for 10 replicates, MAE = mean absolute error of the 6 mutations, SEM = standard error of the mean for 10 replicates per mutation, averaged over the 6 mutations.

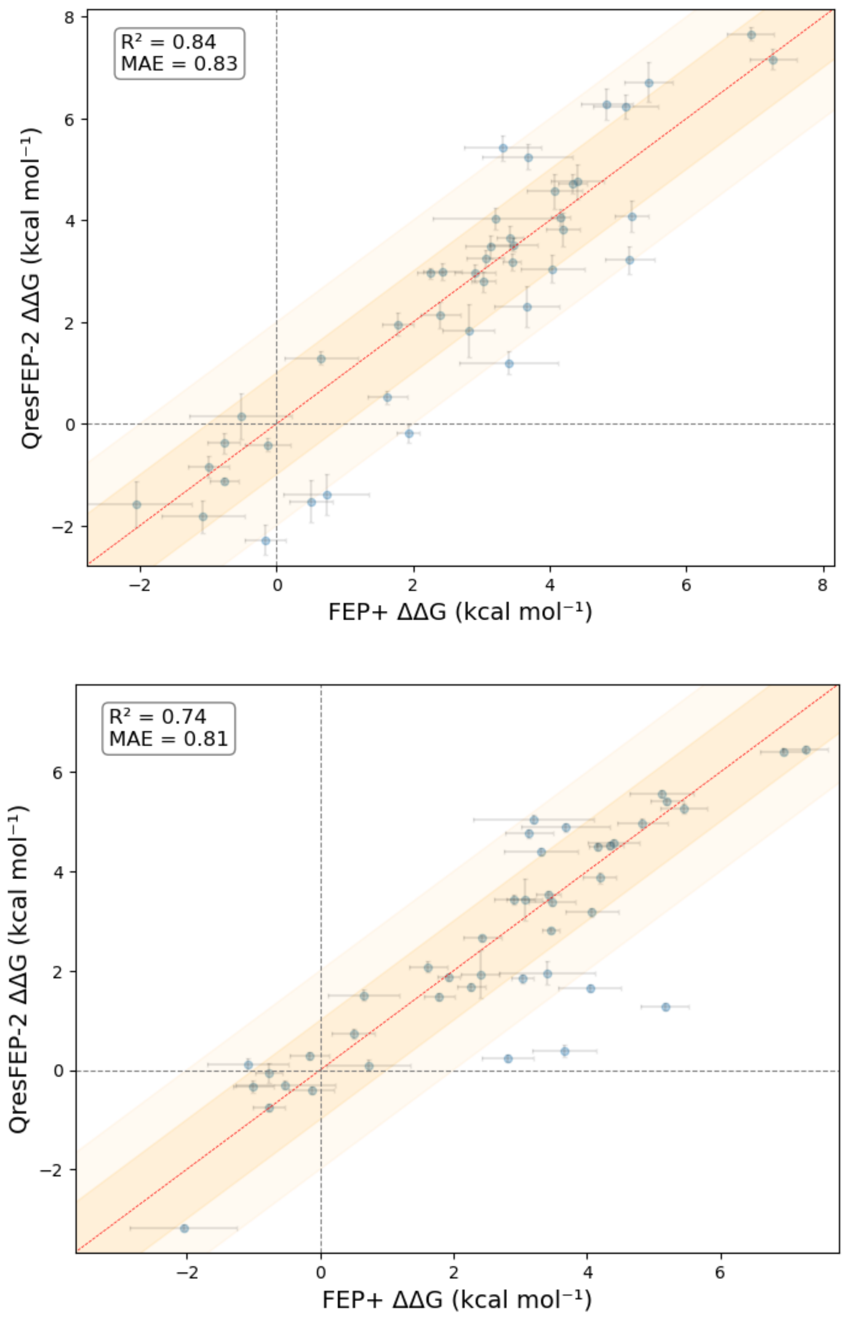

Supplementary Figure 3: Autocorrelation plots for the subset of 43 Ala mutations on T4 lysozyme between the new hybrid-topology QresFEP-2 protocol, and single-topology stepwise annihilation QresFEP-1 protocol (TOP) or the commercial FEP+ protocol (BOTTOM), respectively. Error bars correspond to the SEM values obtained with each method.

Supplementary Table 2. Experimental (exp) and calculated (calc) changes in free energy (ΔΔG) and SEM (kcal mol[-1]) associated with thermal stability after solving the associated thermodynamic cycle (average ± SEM of 10 simulations) for each single-point mutation of T4 lysozyme, using different reference tripeptide models.

mutation	$\Delta\Delta G_{exp}$	$\Delta\Delta G_{calc}$ (H) ZXZ	SEM	$\Delta\Delta G_{calc}$ (J) ZXZ	SEM	$\Delta\Delta G_{calc}$ (D) AXA	SEM	$\Delta\Delta G_{calc}$ (D) GXG	SEM	$\Delta\Delta G_{calc}$ (D) X	SEM
N116A	-0.17	-0.16	0.30	-0.50	0.41	-0.80	0.64	-0.04	0.39	-0.35	0.54
N40A	-0.32	-1.08	0.61	0.69	0.46	-0.84	0.58	-0.15	0.39	-0.08	0.29
N68A	0.05	0.50	0.32	-0.01	0.57	1.46	0.54	1.29	0.66	0.75	0.72
Q105A	0.60	3.40	0.72	2.87	0.80	3.95	0.54	4.07	0.55	4.06	0.56
Q122A	0.24	1.62	0.29	0.86	0.31	0.93	0.32	1.80	0.28	1.03	0.31
Q123A	0.22	0.65	0.54	0.44	0.51	1.04	0.64	1.29	0.62	1.28	0.65
I100A	3.40	2.91	0.30	2.87	0.35	2.91	0.26	2.82	0.34	3.15	0.27
I17A	2.70	4.34	0.21	4.49	0.23	4.84	0.19	4.93	0.18	5.17	0.35
I27A	3.10	3.47	0.35	3.84	0.17	3.36	0.27	3.43	0.27	3.75	0.33
I29A	2.60	4.04	0.47	3.49	0.50	3.93	0.67	4.24	0.65	4.35	0.68
I3A	0.70	3.06	0.27	2.73	0.29	3.54	0.20	3.78	0.20	4.01	0.27
I50A	2.00	1.93	0.17	1.71	0.27	2.18	0.27	2.04	0.34	2.52	0.40
I58A	3.20	4.83	0.38	4.21	0.68	5.11	0.39	5.63	0.45	5.50	0.45
I78A	1.60	3.03	0.17	2.36	0.24	3.01	0.24	3.40	0.27	2.98	0.36
L118A	3.50	5.11	0.48	4.86	0.51	4.80	0.40	5.17	0.42	5.35	0.40
L121A	2.30	3.31	0.56	3.63	0.68	4.27	0.69	5.13	0.69	4.75	0.69
L133A	4.30	3.68	0.66	3.71	0.78	3.88	0.48	4.47	0.45	4.70	0.44
L33A	3.60	4.19	0.25	4.23	0.31	5.24	0.38	5.34	0.42	5.71	0.40
L39A	0.90	1.78	0.23	2.32	0.19	2.65	0.19	2.35	0.44	2.95	0.22
L46A	1.86	3.42	0.19	3.12	0.22	3.67	0.25	3.92	0.24	4.27	0.24
L66A	3.90	5.20	0.24	4.71	0.18	5.19	0.31	5.26	0.30	5.41	0.35
L7A	2.60	5.17	0.36	4.42	0.32	5.02	0.45	5.46	0.47	5.37	0.59
L84A	3.90	7.27	0.34	7.41	0.32	7.35	0.30	7.84	0.33	7.17	0.46
L91A	3.10	4.40	0.39	3.90	0.40	4.84	0.50	4.89	0.49	4.49	0.59
L99A	4.50	6.94	0.34	7.31	0.21	7.06	0.36	6.79	0.48	6.75	0.47
M106A	2.30	3.13	0.36	3.24	0.19	3.61	0.42	4.54	0.42	4.27	0.42
M120A	0.20	2.81	0.38	1.81	0.47	2.28	0.57	2.73	0.58	2.72	0.57
M6A	1.90	3.20	0.91	2.54	0.71	3.59	0.56	4.09	0.57	3.92	0.63
F104A	3.10	4.07	0.40	2.70	0.38	4.40	0.51	4.72	0.48	4.86	0.49
F153A	3.80	5.45	0.35	4.73	0.41	4.98	0.38	5.80	0.45	5.59	0.37
F67A	1.90	3.66	0.48	3.60	0.60	4.83	0.66	5.53	0.62	5.33	0.63
S117A	-1.27	-2.05	0.81	-1.47	0.51	-2.23	0.96	-1.81	1.01	-3.05	0.97
S44A	-0.34	-0.52	0.75	-0.80	0.48	0.09	0.86	-0.53	0.85	0.30	0.78
T115A	0.14	0.73	0.63	0.54	0.55	0.62	0.38	0.86	0.31	0.87	0.50
T157A	0.50	-1.00	0.30	-0.95	0.47	-0.46	0.41	-0.29	0.40	-0.32	0.41
T59A	1.50	2.40	0.29	2.17	0.27	2.51	0.31	2.38	0.28	2.12	0.31
V103A	1.91	2.26	0.21	2.41	0.26	2.64	0.15	2.99	0.17	3.83	0.29
V111A	1.30	-0.13	0.34	-0.18	0.24	-0.90	0.31	-0.71	0.32	-0.40	0.32
V131A	-0.39	-0.77	0.21	-0.88	0.18	-0.76	0.13	-0.26	0.17	-0.02	0.18
V149A	2.87	4.16	0.14	3.93	0.22	4.02	0.31	4.21	0.36	4.39	0.33
V71A	1.50	3.45	0.13	3.20	0.21	3.16	0.16	3.11	0.14	3.79	0.21
V87A	1.70	2.43	0.29	2.51	0.21	2.64	0.21	3.00	0.21	3.05	0.21
V94A	1.80	-0.77	0.24	-0.75	0.17	-0.91	0.30	-0.86	0.30	-0.66	0.30
N55G	0.60	1.29	0.80	1.69	0.67	-0.31	0.82	0.15	0.75	1.86	0.85
Q105G	3.11	5.05	1.08	4.22	0.57	5.51	0.53	6.64	0.51	8.00	0.49
I3G	2.10	4.88	0.45	5.52	0.38	5.19	0.50	5.51	0.65	7.12	0.49
I3T	2.30	5.42	0.61	4.96	0.46	5.43	0.44	5.50	0.43	5.79	0.45
I3V	0.40	0.64	0.20	0.18	0.31	0.49	0.24	0.53	0.24	0.75	0.25
L99G	6.30	9.64	0.41	10.00	0.43	9.75	0.39	10.62	0.43	10.29	0.61
F153L	-0.30	-0.04	0.30	0.02	0.30	0.29	0.45	-0.45	0.49	0.05	0.51
S44G	0.53	0.74	0.52	0.67	0.45	0.97	0.34	0.79	0.57	1.49	0.74
T151S	-0.39	-1.57	0.24	-1.55	0.25	-0.98	0.59	-0.88	0.62	-0.61	0.58
T152S	2.60	1.30	0.64	1.47	0.77	0.87	0.50	1.31	0.60	0.98	0.44
T157G	1.10	-0.55	0.27	0.48	0.49	0.97	0.49	0.80	0.32	1.86	0.29
T157S	0.66	-1.90	0.29	-1.21	0.40	-0.57	0.40	-0.81	0.42	-0.62	0.36
T157V	1.20	-0.28	0.34	-0.94	0.32	0.65	0.38	0.79	0.34	0.67	0.31
T26S	-0.57	5.51	0.70	4.54	0.39	6.61	0.36	6.82	0.38	7.36	0.36
T59G	1.60	1.54	0.44	1.77	0.51	2.65	0.29	3.07	0.29	3.93	0.19
T59S	0.20	-0.64	0.37	-0.73	0.42	-0.17	0.58	-0.45	0.49	0.10	0.51
T59V	1.50	5.28	0.20	5.02	0.22	5.19	0.32	4.68	0.19	4.73	0.21
Y25G	4.55	4.77	0.43	4.14	0.62	4.85	0.46	5.31	0.48	7.15	0.47
V131G	0.68	0.50	0.18	1.15	0.39	0.49	0.26	0.96	0.18	2.34	0.19
V131T	0.12	-0.51	1.36	1.22	1.33	-0.27	1.37	-0.72	1.42	-0.76	1.33
V149T	2.80	-0.72	0.73	-0.39	0.60	-1.22	0.53	-0.92	0.54	-1.10	0.62
V75T	1.30	2.94	0.34	2.53	0.26	3.28	0.29	3.10	0.26	3.57	0.26
V87T	1.60	2.48	0.78	3.69	0.59	3.52	0.71	3.74	0.70	3.81	0.76

Protocol	*n*	MAE (kcal mol[-1])	Accuracy (%)	MCC	R^2	ρ	τ
(H) ZXZ	66	1.41	84.9	0.55	0.59	0.73	0.58

(J) ZXZ	66	1.28	83.3	0.40	0.60	0.74	0.57
(H) AXA	66	1.52	81.8	0.38	0.54	0.70	0.54
(H) GXG	66	1.62	81.8	0.31	0.55	0.72	0.55
(H) X	66	1.65	86.4	0.52	0.54	0.72	0.55

Supplementary Table 3. Experimental and calculated changes in free energy for Ribonuclease Barnase.

mutation	$\Delta\Delta G_{exp}$	$\Delta\Delta G_{calc}$ (H) ZXZ	SEM	$\Delta\Delta G_{calc}$ (D) ZXZ	SEM	$\Delta\Delta G_{calc}$ (D) AXZ	SEM	$\Delta\Delta G_{calc}$ (D) GXG	SEM	$\Delta\Delta G_{calc}$ (D) X	SEM
A32N	0.70	0.81	0.14	0.85	0.26	0.72	0.28	0.82	0.24	0.48	0.26
A32C	1.00	0.48	0.22	0.00	0.21	0.14	0.21	0.24	0.20	0.18	0.19
A32Q	0.50	0.09	0.13	0.04	0.17	0.25	0.20	0.32	0.35	-0.33	0.46
A32G	0.90	0.76	0.10	0.59	0.10	0.87	0.20	1.42	0.13	2.55	0.15
A32H	0.80	0.58	0.31	0.74	0.26	0.75	0.29	0.34	0.45	0.51	0.25
A32I	0.80	1.45	0.16	1.11	0.23	1.17	0.14	0.55	0.17	0.51	0.20
A32L	0.30	0.13	0.21	0.44	0.20	0.67	0.14	0.40	0.25	-0.29	0.43
A32M	0.30	0.91	0.16	0.37	0.26	0.31	0.29	-0.34	0.46	-0.52	0.49
A32F	0.70	0.63	0.27	0.57	0.23	0.64	0.21	-0.25	0.46	-0.83	0.59
A32S	0.40	0.44	0.35	0.69	0.31	0.51	0.22	0.67	0.37	0.54	0.33
A32T	0.80	0.18	0.44	0.84	0.32	1.02	0.30	0.83	0.29	1.24	0.29
A32W	1.00	0.11	0.37	0.43	0.36	0.43	0.42	0.11	0.38	-1.38	0.92
A32Y	0.80	0.27	0.22	1.38	0.27	1.03	0.26	-1.03	0.80	0.13	0.61
A32V	0.90	1.41	0.12	1.25	0.16	1.45	0.11	1.01	0.15	0.87	0.17
N23A	2.20	3.89	0.39	4.68	0.47	4.18	0.71	5.42	0.45	5.22	0.44
N58A	2.70	3.75	0.69	3.89	0.60	3.94	0.57	4.56	0.56	5.17	0.62
N5A	1.90	2.31	0.28	2.41	0.43	2.50	0.35	2.22	0.47	2.31	0.47
N77A	1.60	0.21	0.23	0.31	0.29	0.18	0.55	0.61	0.49	0.84	0.34
N84A	2.00	3.26	0.53	4.02	0.34	3.76	0.44	3.94	0.45	4.27	0.29
Q104A	0.20	-0.14	0.29	0.22	0.24	-0.07	0.30	0.48	0.24	0.50	0.22
Q15A	0.20	-0.46	0.40	0.32	0.45	0.01	0.33	0.07	0.44	0.72	0.42
Q15G	1.60	0.83	0.44	0.84	0.48	0.91	0.50	1.30	0.41	3.39	0.37
Q15I	-1.00	-1.32	0.46	-2.88	0.52	-2.84	0.38	-2.63	0.48	-3.24	0.36
Q31A	-0.10	-1.12	0.31	-0.72	0.29	-0.67	0.33	-0.51	0.39	-0.60	0.36
Q31G	0.98	0.64	0.48	0.41	0.42	0.42	0.51	0.93	0.40	2.13	0.39
Q31S	0.20	-0.88	0.34	-1.36	0.50	-0.79	0.53	-0.91	0.57	-0.71	0.53
G34A	3.11	1.83	0.27	1.10	0.11	3.12	0.24	5.09	0.17	1.56	0.08
G34N	2.74	1.08	0.25	1.14	0.46	2.53	0.57	1.75	0.40	1.21	0.49
G34H	2.65	1.80	0.28	0.19	0.42	2.33	0.30	0.94	0.29	-0.20	0.31
G34S	3.20	1.35	0.76	1.53	0.83	4.25	0.77	2.94	0.72	3.30	0.54
G34T	3.37	3.29	0.43	2.00	0.48	3.71	0.31	3.84	0.21	2.71	0.26
G65S	-0.50	-0.84	0.80	-2.85	0.81	-1.35	0.78	-1.50	0.87	-2.60	0.79
H18A	1.85	0.94	0.34	1.07	0.28	1.69	0.26	1.95	0.48	1.71	0.21
H18N	1.68	0.22	0.29	0.63	0.30	1.17	0.27	1.20	0.24	1.28	0.25
H18Q	1.60	0.76	0.40	0.78	0.43	1.01	0.53	0.88	0.44	0.85	0.47
H18G	0.70	0.87	0.30	0.47	0.28	-0.86	0.25	0.02	0.44	1.09	0.26
H18S	2.28	1.60	0.48	0.99	0.48	1.57	0.44	1.93	0.52	1.86	0.48
I109A	2.10	3.66	0.30	3.74	0.39	4.35	0.58	3.61	0.30	4.83	0.40
I109V	0.80	1.68	0.19	2.47	0.28	2.33	0.30	2.72	0.27	2.60	0.25
I25A	3.49	4.68	0.30	5.01	0.18	5.37	0.24	5.35	0.29	5.71	0.26
I25V	1.10	1.93	0.13	1.58	0.16	1.40	0.13	1.41	0.14	1.59	0.14
I4A	1.40	3.19	0.20	2.66	0.39	2.65	0.32	3.29	0.34	3.41	0.32
I4V	0.60	0.88	0.12	0.55	0.18	0.80	0.12	0.73	0.11	0.86	0.11
I51A	4.69	4.37	0.54	4.51	0.56	4.35	0.55	4.79	0.57	4.90	0.57
I51V	1.80	2.09	0.17	2.15	0.25	1.92	0.24	1.82	0.25	1.73	0.47
I55A	1.10	2.23	0.23	2.53	0.24	2.59	0.21	3.18	0.24	3.14	0.26
I55G	3.10	3.69	0.31	3.15	0.27	3.27	0.26	4.20	0.33	5.35	0.28
I55T	0.60	0.98	0.52	2.66	0.58	2.07	0.73	3.08	0.57	2.81	0.60
I55V	0.30	0.40	0.11	0.33	0.17	0.35	0.11	0.43	0.14	0.47	0.12
I76A	1.90	-3.35	0.61	-2.29	0.58	-1.68	0.58	-1.26	0.58	-1.29	0.58
I76V	0.80	0.67	0.24	0.27	0.25	0.32	0.26	0.42	0.28	0.41	0.26
I88A	3.99	4.92	0.26	5.47	0.21	5.25	0.21	5.59	0.21	5.82	0.19
I88G	7.39	7.30	0.28	7.23	0.32	7.36	0.31	7.83	0.49	9.14	0.49
I88L	0.30	-0.14	0.44	-0.35	0.21	-0.93	0.21	-0.63	0.24	-1.19	0.53
I88V	1.30	2.19	0.15	1.82	0.19	2.13	0.18	2.29	0.15	2.28	0.18
I96A	3.20	3.63	0.29	4.35	0.37	4.42	0.41	4.65	0.38	4.71	0.39
I96G	5.69	5.02	0.61	6.37	0.30	6.82	0.28	7.70	0.32	8.64	0.41
I96V	0.90	0.90	0.30	1.12	0.22	1.01	0.19	0.95	0.20	1.13	0.20
L14A	4.29	4.37	0.34	4.53	0.42	4.72	0.45	4.74	0.48	5.41	0.43
L33Q	1.30	0.77	0.41	1.19	0.45	1.36	0.37	1.63	0.38	1.42	0.45
L89G	6.99	6.91	0.58	6.27	0.29	6.88	0.42	7.84	0.52	9.11	0.35
L89T	2.50	1.99	0.69	2.65	0.50	2.93	0.32	3.65	0.51	3.21	0.53
L89V	0.30	0.59	0.32	-0.88	0.34	-0.75	0.34	-0.69	0.37	-0.61	0.35
L96G	4.69	4.81	0.42	5.11	0.29	5.09	0.30	6.34	0.41	6.98	0.52
F7L	4.59	2.45	0.36	2.66	0.32	2.57	0.41	2.98	0.31	2.75	0.29

S28A	-0.41	-0.60	0.40	-0.66	0.31	0.22	0.45	-0.50	0.29	-0.10	0.38
S29G	0.45	-0.54	0.54	0.40	0.61	0.57	0.56	0.86	0.54	2.44	0.58
S85A	0.12	-1.57	0.42	-1.29	0.41	-1.37	0.33	-1.30	0.44	-1.34	0.48
S91A	1.90	1.32	0.26	0.15	0.41	0.07	0.34	0.26	0.56	0.62	0.35
S92A	2.79	4.23	0.38	3.45	0.24	4.01	0.34	3.69	0.30	3.77	0.26
T100G	2.79	0.46	0.38	-0.26	0.23	-0.09	0.26	0.61	0.31	1.64	0.21
T105V	2.20	2.48	0.52	4.32	0.68	3.65	0.50	3.40	0.52	3.73	0.58
T16A	0.27	-0.07	0.52	-0.30	0.67	-0.82	0.35	-0.09	0.39	-0.06	0.51
T16G	1.66	0.44	0.36	0.13	0.62	0.52	0.68	1.03	0.75	1.43	0.71
T16S	1.70	0.53	0.64	-0.60	0.58	-0.49	0.63	-0.86	0.47	-1.11	0.49
T26A	1.90	1.80	0.34	1.94	0.33	1.73	0.34	1.82	0.32	2.13	0.32
T26N	1.29	2.82	0.53	3.60	0.46	3.34	0.41	3.55	0.41	3.81	0.43
T26Q	1.72	1.54	0.38	1.92	0.34	1.94	0.45	1.53	0.38	1.28	0.38
T26G	1.50	2.25	0.52	2.13	0.57	2.33	0.63	3.04	0.67	3.66	0.55
T26S	0.56	0.79	0.38	0.14	0.50	-0.17	0.46	0.53	0.54	-0.06	0.46
T26V	2.31	0.70	0.53	2.00	0.41	2.87	0.39	3.06	0.39	2.33	0.39
T6A	2.10	0.83	0.37	0.35	0.19	0.79	0.19	0.95	0.17	1.10	0.18
T6N	1.27	0.87	0.63	1.01	0.81	1.42	0.76	1.68	0.75	1.67	0.74
T6Q	1.87	0.17	0.33	-0.20	0.33	-0.23	0.56	-0.20	0.29	-0.14	0.32
T6G	1.20	0.04	0.26	-0.63	0.29	0.09	0.32	0.72	0.23	1.44	0.21
T6H	2.65	0.27	0.25	0.44	0.19	0.80	0.23	0.96	0.20	1.06	0.20
T6S	0.22	0.28	0.39	0.08	0.35	0.04	0.33	0.67	0.33	0.42	0.29
T6V	-0.30	1.21	0.42	0.76	0.57	0.66	0.49	0.98	0.60	0.98	0.62
T99V	2.70	1.20	0.58	2.18	0.55	1.34	0.80	1.72	0.76	1.40	0.65
W94L	1.59	1.53	0.30	0.46	0.43	1.78	0.42	1.47	0.44	1.46	0.47
W94F	1.06	1.55	0.37	1.46	0.25	0.72	0.31	1.08	0.35	1.58	0.29
W94Y	1.27	1.35	0.24	1.79	0.51	1.09	0.52	0.57	0.50	0.93	0.49
Y103F	0.00	1.78	0.35	2.67	0.47	2.20	0.49	2.58	0.51	2.65	0.47
Y13A	3.29	3.98	0.37	3.71	0.39	3.65	0.33	3.53	0.45	3.81	0.37
Y13G	6.39	5.66	0.60	6.16	0.84	5.89	0.76	6.21	1.00	8.21	0.76
Y13F	0.41	0.54	0.28	1.17	0.26	0.93	0.24	1.49	0.29	1.41	0.22
Y17A	2.00	1.84	0.26	1.12	0.37	1.69	0.41	1.91	0.56	2.28	0.40
Y17G	4.02	3.12	0.42	4.47	0.35	5.25	0.38	5.83	0.42	7.23	0.34
Y17F	0.30	0.15	0.14	0.18	0.16	0.28	0.21	0.39	0.29	0.53	0.20
Y17S	2.57	1.72	0.37	2.26	0.45	2.29	0.46	2.61	0.50	3.43	0.45
Y24F	0.00	1.05	0.17	2.09	0.25	1.77	0.19	1.44	0.29	1.96	0.21
Y78F	1.40	0.40	0.35	0.51	0.29	0.61	0.25	0.30	0.26	0.72	0.19
Y97G	6.59	9.20	0.43	9.13	0.64	8.92	0.59	10.26	0.67	11.41	0.61
V10A	3.39	4.35	0.14	4.27	0.14	4.32	0.11	4.59	0.11	4.66	0.09
V10T	2.50	-0.60	0.11	-0.83	0.20	-0.58	0.44	-0.80	0.12	-0.32	0.69
V36A	1.30	1.11	0.15	0.93	0.17	1.19	0.15	1.36	0.10	1.37	0.17
V36T	1.10	-0.72	0.48	-2.65	0.54	-2.89	0.54	-2.44	0.62	-2.48	0.54
V45A	1.80	1.62	0.24	1.45	0.39	1.30	0.28	1.63	0.27	1.69	0.29
V45T	2.40	0.95	0.81	1.34	0.74	1.43	0.71	1.63	0.66	1.56	0.71

Protocol	*n*	MAE (kcal mol^{-1})	Accuracy (%)	MCC	R^2	ρ	τ
(H) ZXZ	109	0.83	88.1	0.35	0.66	0.70	0.54
(H) AXA	109	0.81	87.2	0.33	0.70	0.74	0.59
(H) GXG	109	0.86	82.6	0.26	0.72	0.77	0.60
(H) X	109	1.09	80.7	0.17	0.67	0.69	0.52

Mutation	$\Delta\Delta G_{exp}$ (kcal mol^{-1})	PyMOL |Error|	AlphaFold2 |Error|	AlphaFold3 |Error|
H18N	1.68	**0.52**	1.19	0.86
I55T	0.60	0.55	**0.03**	0.57
I88L	0.30	2.50	1.89	**1.30**
L33Q	1.30	1.03	**0.57**	1.49
F7L	4.59	1.82	**1.57**	2.92
T26N	1.29	1.94	**0.22**	0.27
T26S	0.56	0.81	0.57	**0.50**
T6N	1.27	**0.82**	1.22	1.72
T6Q	1.87	1.86	**1.18**	1.50
T6H	2.65	**1.72**	3.22	3.73
T6S	0.22	0.83	0.34	**0.00**
T79V	-0.30	**0.70**	1.87	1.04
T99V	2.70	1.72	2.43	**0.52**
W94L	1.59	0.52	1.26	**0.08**
	Average	1.24	1.25	1.18

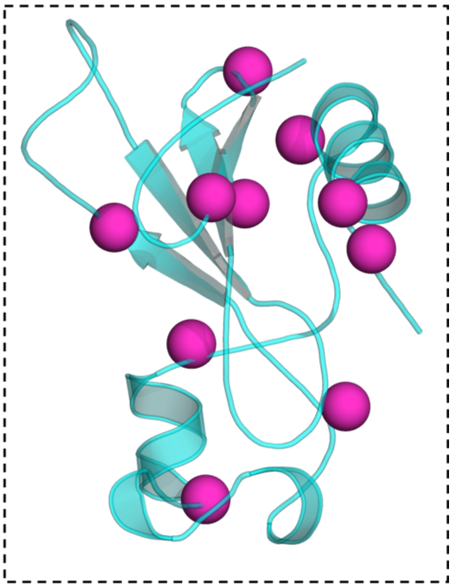

Supplementary Figure 4. Comparison of the unassigned errors associated to FEP calculations starting from PyMOL, AlphaFold2 and AlphaFold3 generated mutant conformations. The lowest error registered for each mutation is indicated in bold. The distribution of the subset of 14 mutations considered is shown on the structure of ribonuclease Barnase as magenta spheres.

Supplementary Figure 5. Experimental vs calculated shifts in thermal stability, expressed as $\Delta\Delta G$ (kcal mol^{-1}), for T4 lysozyme.

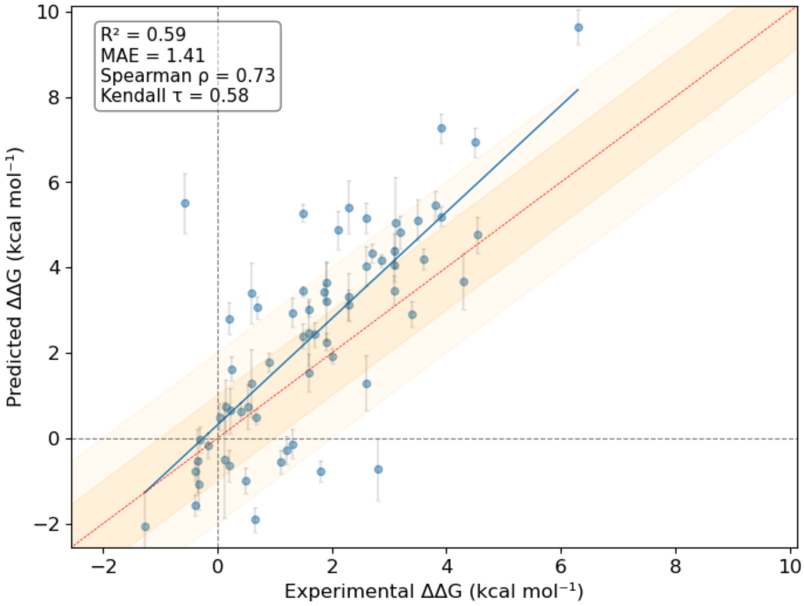

Supplementary Figure 6. Experimental vs calculated shifts in thermal stability, expressed as $\Delta\Delta G$ (kcal mol^{-1}), for Barnase ribonuclease.

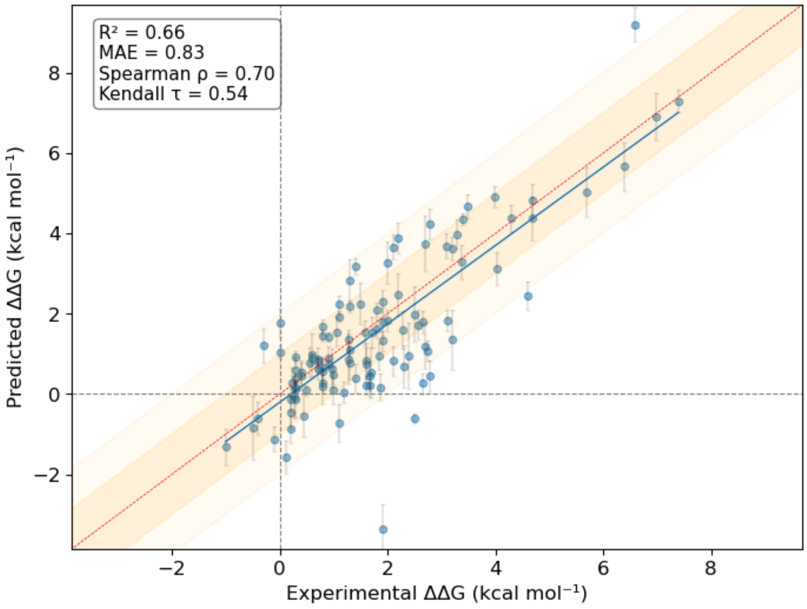

Supplementary Figure 7 Experimental vs calculated shifts in thermal stability, expressed as ΔΔ*G* (kcal mol^{-1}), for Staphylococcal nuclease.

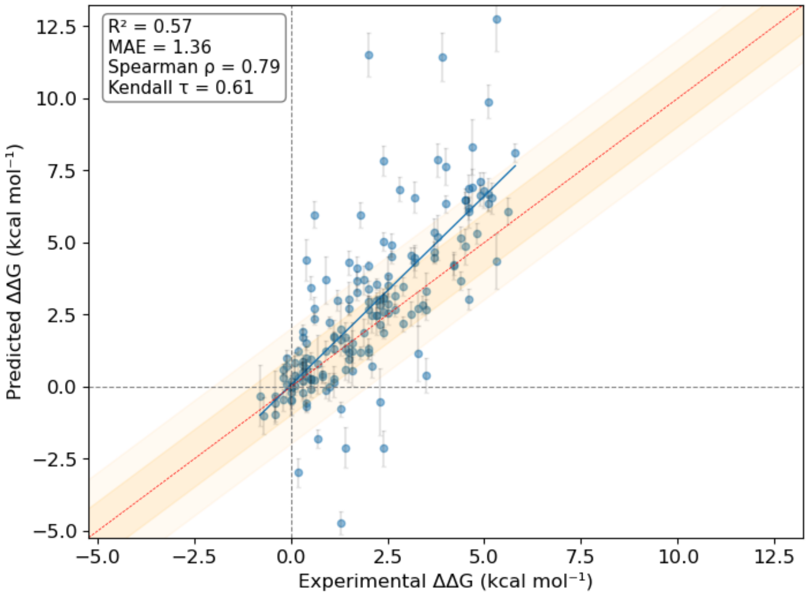

Supplementary Figure 8 Experimental vs calculated shifts in thermal stability, expressed as ΔΔ*G* (kcal mol^{-1}), for Chymotrypsin inhibitor 2.

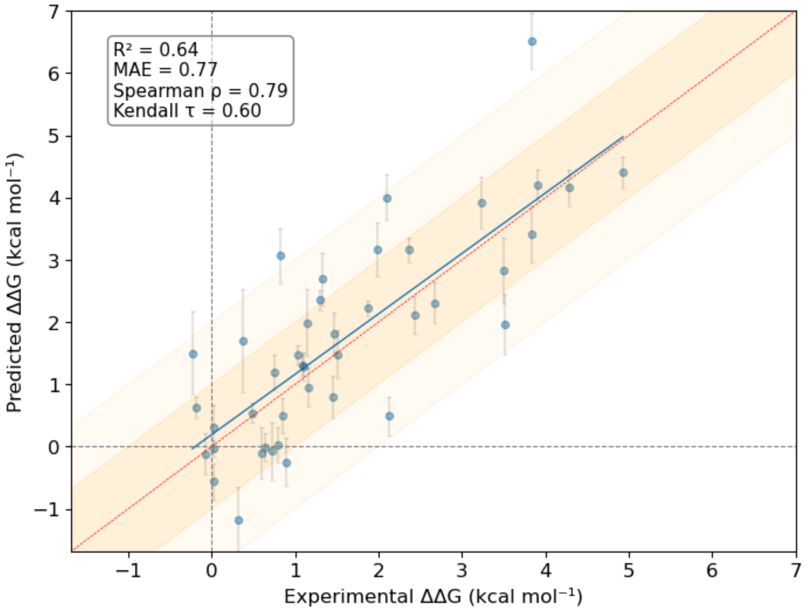

Supplementary Figure 9 Experimental vs calculated shifts in thermal stability, expressed as $\Delta\Delta G$ (kcal mol^{-1}), for Protein L, B1 domain

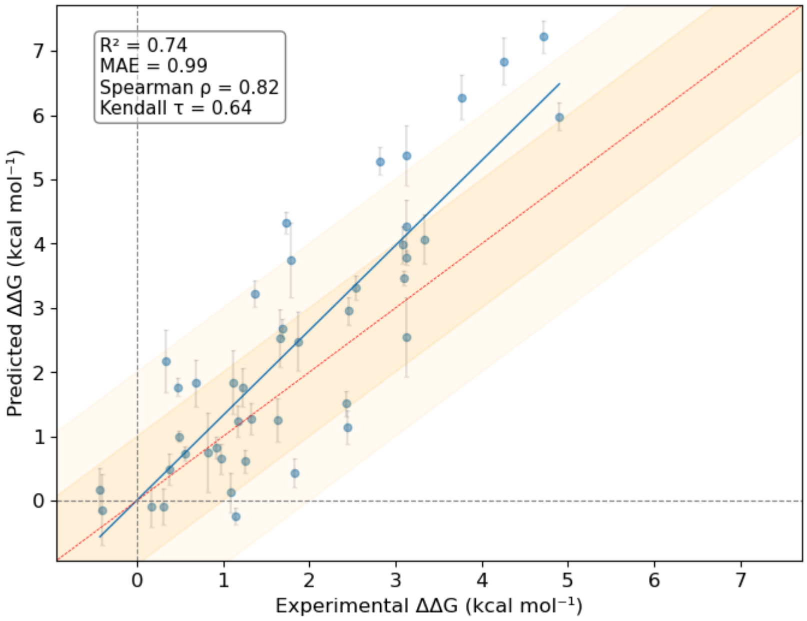

Supplementary Figure 10 Experimental vs calculated shifts in thermal stability, expressed as $\Delta\Delta G$ (kcal mol^{-1}), for c-SRC tyrosine kinase

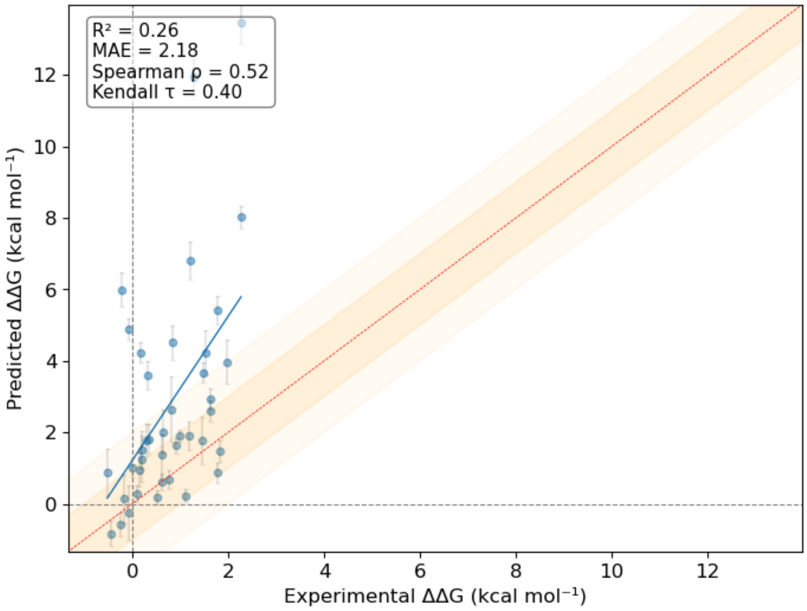

Supplementary Figure 11 Experimental vs calculated shifts in thermal stability, expressed as $\Delta\Delta G$ (kcal mol^{-1}), for Human Lysozyme

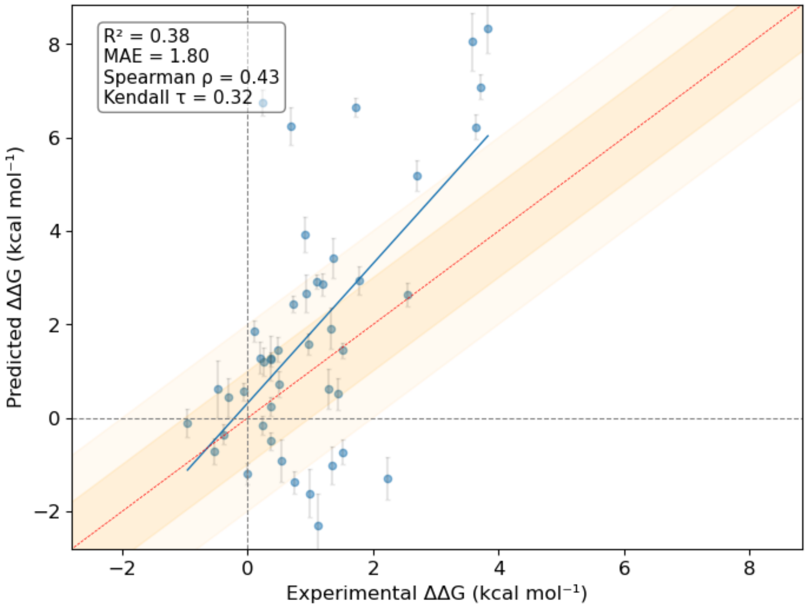

Supplementary Figure 12 Experimental vs calculated shifts in thermal stability, expressed as $\Delta\Delta G$ (kcal mol^{-1}), for Fibronectin III domain.

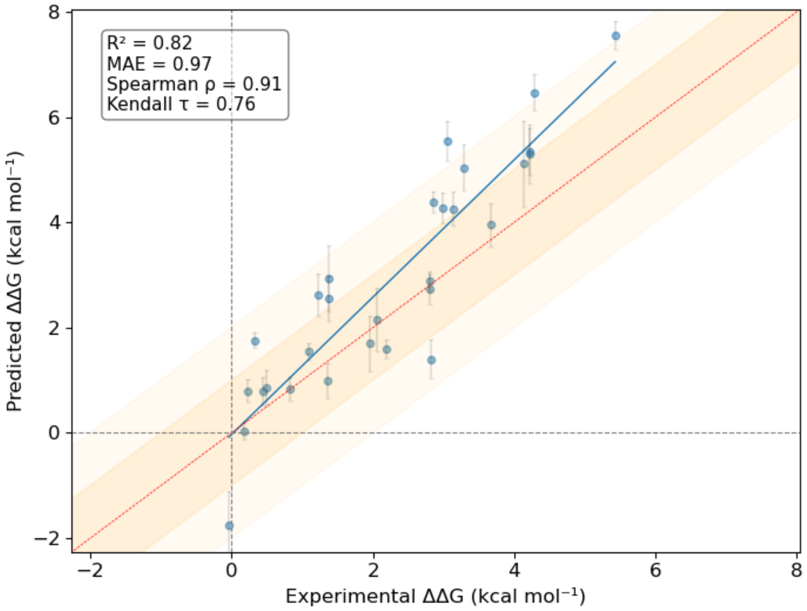

Supplementary Figure 13 Experimental vs calculated shifts in thermal stability, expressed as ΔΔG (kcal mol[-1]), for Trypsin inhibitor.

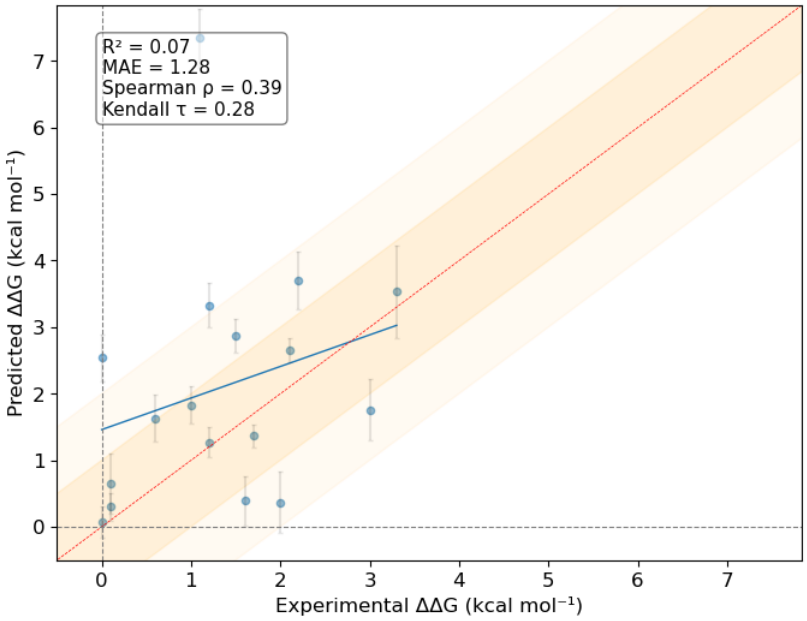

Supplementary Figure 14 Experimental vs calculated shifts in thermal stability, expressed as ΔΔG (kcal mol[-1]), for FK506 binding protein

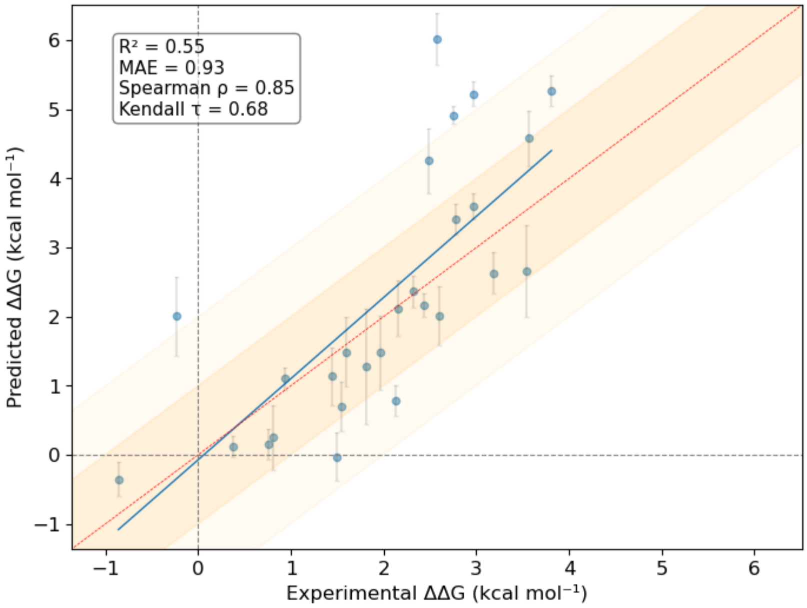

Supplementary Table 4. Experimental and calculated changes in free energy for Staphylococcal nuclease.

mutation	$\Delta\Delta G_{exp}$	$\Delta\Delta G_{calc}$ (H) ZXZ	SEM	mutation	$\Delta\Delta G_{exp}$	$\Delta\Delta G_{calc}$ (H) ZXZ	SEM	mutation	$\Delta\Delta G_{exp}$	$\Delta\Delta G_{calc}$ (H) ZXZ	SEM
A102G	1.30	1.64	0.15	*L125A*	4.90	6.62	0.36	*T82A*	0.90	-0.11	0.28
A109G	1.00	2.23	0.21	*L137A*	2.30	2.16	0.24	*T82G*	2.00	1.30	0.36
A112G	0.00	-0.44	0.52	*L137G*	4.60	3.03	0.36	*T82V*	-0.20	-0.45	0.38
A12G	2.40	3.06	0.17	*L14A*	2.30	2.82	0.25	*Y113A*	0.00	0.19	0.20
A130G	1.10	1.31	0.13	*L14G*	3.70	4.66	0.37	*Y113F*	0.00	0.87	0.26
A132G	3.70	4.46	0.14	*L25A*	2.70	3.17	0.34	*Y113G*	0.30	0.30	0.17
A17G	0.30	1.73	0.26	*L25G*	4.50	4.87	0.62	*Y113L*	-0.20	-0.21	0.42
A58G	2.60	4.50	0.21	*L36A*	3.50	3.33	0.63	*Y115A*	0.30	0.21	0.62
A60G	1.40	1.30	0.18	*L36G*	5.30	4.37	0.96	*Y115F*	0.10	0.78	1.03
A69G	2.00	1.20	0.26	*L37A*	1.70	4.13	0.40	*Y115G*	0.70	0.74	0.24
A90G	2.00	2.94	0.20	*L37G*	3.80	7.86	0.57	*Y115L*	0.30	0.40	0.68
A94G	2.40	1.89	0.20	*L38A*	1.70	3.66	0.34	*Y27A*	2.80	6.84	0.42
N100A	5.20	6.55	0.54	*L38G*	0.60	5.97	0.45	*Y27F*	0.60	9.89	0.59
N100G	5.10	6.66	0.56	*L7A*	1.60	0.54	0.49	*Y27G*	5.10	4.30	0.42
N118A	2.10	0.71	0.41	*L7G*	1.50	0.95	0.78	*Y27L*	1.50	2.35	0.20
N118G	1.90	1.86	0.48	*L89A*	2.60	4.92	0.40	*Y54A*	2.20	2.49	0.68
N119A	1.30	-0.77	0.26	*L89G*	3.20	6.57	0.53	*Y54F*	0.50	3.70	0.50
N119G	1.30	-4.73	0.39	*M26A*	1.50	2.70	0.20	*Y54G*	1.90	2.83	0.32
N138A	1.10	1.71	0.19	*M26G*	2.20	3.56	0.21	*Y54L*	3.40	0.27	0.11
N138G	-0.10	0.98	0.28	*M32A*	1.70	3.26	0.26	*Y85A*	0.40	0.98	0.42
N68A	0.50	0.24	0.39	*M32G*	2.40	5.04	0.31	*Y85F*	0.00	-0.02	0.48
N68G	0.50	-0.07	0.30	*M65A*	2.00	4.18	0.16	*Y85G*	1.00	0.04	0.22
Q106A	-0.10	0.71	0.35	*M65G*	4.60	6.07	0.54	*Y85L*	0.10	-0.01	0.74
Q106G	1.50	3.05	0.58	*M98A*	4.60	6.18	0.37	*Y91A*	5.30	12.75	1.10
Q123A	0.40	0.37	0.28	*M98G*	4.50	6.47	0.42	*Y91F*	2.40	11.43	0.83
Q123G	0.60	0.25	0.38	*F34A*	3.70	5.35	0.47	*Y91L*	3.90	7.84	0.51
Q131A	0.20	1.25	0.16	*F61A*	2.30	3.02	0.42	*Y93F*	2.00	11.51	0.76
Q131G	2.40	3.08	0.31	*F61G*	4.80	5.32	0.36	*Y93G*	7.50	6.47	0.27
Q30A	0.30	0.53	0.36	*F76A*	4.00	7.63	0.63	*Y93L*	4.50	3.40	0.28
Q30G	0.90	1.25	0.35	*F76G*	4.70	8.30	0.96	*V104A*	2.90	2.19	0.26
Q80A	0.10	0.84	0.37	*S128A*	-0.70	-1.01	0.65	*V104T*	2.50	3.50	0.36
Q80G	1.40	1.73	0.49	*S128G*	1.60	1.24	0.71	*V111A*	4.20	4.23	0.35
H121A	3.10	-0.58	0.28	*S59A*	-0.40	-0.33	0.88	*V111G*	4.90	7.11	0.31
H121G	4.20	0.29	0.34	*S59G*	1.10	1.74	0.58	*V111T*	2.30	-0.52	1.16
H124A	-0.40	4.55	0.28	*T120A*	1.20	2.99	0.36	*V114A*	0.00	-0.06	0.19
H124G	0.50	4.20	0.47	*T120G*	2.10	2.47	0.60	*V114G*	0.20	0.33	0.48
H46A	0.50	3.42	0.40	*T120V*	1.80	5.94	0.44	*V114T*	0.30	1.91	0.26
H46G	0.40	4.40	0.72	*T13A*	0.70	-1.81	0.31	*V23A*	2.90	3.47	0.33
H8A	0.40	-0.58	0.29	*T13G*	1.10	0.16	0.54	*V23G*	5.60	6.08	0.46
H8G	0.80	0.34	0.23	*T13V*	0.40	-0.68	0.16	*V23T*	3.20	4.31	0.51
I139A	3.50	2.67	0.37	*T22A*	1.60	1.53	0.70	*V39A*	2.20	2.98	0.31
I139G	4.40	3.69	0.32	*T22G*	2.40	2.61	0.66	*V39G*	4.70	6.91	0.64
I139V	1.50	1.18	0.27	*T22V*	0.90	3.70	0.82	*V39T*	1.30	2.01	0.92
I15A	2.70	2.67	0.22	*T33A*	1.40	0.60	0.38	*V51A*	0.30	-0.20	0.30
I15G	3.30	2.71	0.61	*T33G*	2.50	2.56	0.40	*V51G*	0.40	1.52	0.23
I15V	0.80	0.43	0.28	*T33V*	-0.40	-0.98	0.29	*V51T*	-0.20	0.58	0.20
I18A	2.50	2.87	0.38	*T41A*	0.00	-0.50	0.52	*V66A*	2.20	2.47	0.30
I18G	2.50	3.82	0.70	*T41G*	2.00	2.69	0.48	*V66G*	4.40	5.17	0.38
I18V	1.10	0.26	0.20	*T41V*	-0.80	-0.31	1.05	*V66T*	1.40	-2.11	0.71
I72A	5.10	6.37	0.28	*T44A*	0.40	0.62	0.34	*V74A*	3.10	2.53	0.43
I72V	1.80	1.18	0.23	*T44G*	0.60	2.71	0.40	*V74T*	3.80	5.18	0.78
I92A	4.00	6.34	0.31	*T44V*	-0.10	0.09	0.45	*V99A*	3.20	4.47	0.45
I92V	0.50	0.97	0.24	*T62A*	2.40	-2.14	0.62	*V99G*	5.00	6.81	0.61
L103A	4.60	6.86	0.49	*T62G*	3.50	0.39	0.59	*V99T*	3.30	1.15	0.95
L108A	5.80	8.11	0.32	*T62V*	0.20	-2.98	0.50				

n	MAE (kcal mol^{-1})	Accuracy (%)	MCC	R^2	ρ	τ
164	1.36	88.4	0.48	0.57	0.79	0.61

Supplementary Table 5. Experimental and calculated changes in free energy for Chymotrypsin inhibitor 2.

mutation	$\Delta\Delta G_{exp}$	$\Delta\Delta G_{calc}$ (H) ZXZ	SEM	*mutation*	$\Delta\Delta G_{exp}$	$\Delta\Delta G_{calc}$ (H) ZXZ	SEM	*mutation*	$\Delta\Delta G_{exp}$	$\Delta\Delta G_{calc}$ (H) ZXZ	SEM
A35G	1.09	1.31	0.17	*L27A*	2.68	2.31	0.33	*T58A*	0.72	-0.07	0.46
A77G	1.88	2.22	0.12	*L40A*	1.33	2.69	0.42	*V38A*	0.49	0.54	0.16
N75A	0.83	3.07	0.44	*L40G*	2.10	4.00	0.37	*V53A*	0.64	-0.01	0.23
Q41A	0.02	-0.56	0.29	*L51A*	2.37	3.16	0.20	*V53G*	2.43	2.12	0.30
Q41G	0.60	-0.11	0.41	*L68A*	3.84	3.41	0.45	*V53T*	1.03	1.47	0.16
I39V	1.30	2.35	0.16	*F69A*	3.84	6.51	0.45	*V57A*	1.47	1.82	0.34
I48A	3.90	4.20	0.25	*S31A*	0.89	-0.25	0.38	*V66A*	4.93	4.40	0.25
I48V	1.11	1.29	0.22	*S31G*	0.80	0.02	0.28	*V70A*	1.98	3.17	0.43
I49A	2.12	0.49	0.31	*T22A*	0.85	0.50	0.28	*V79A*	1.51	1.48	0.39
I49G	3.52	1.97	0.49	*T22G*	1.16	0.95	0.31	*V79G*	3.24	3.92	0.41
I49V	-0.08	-0.12	0.33	*T22V*	0.32	-1.18	0.52	*V79T*	0.38	1.70	0.82
I56A	0.03	-0.03	0.13	*T55A*	-0.23	1.50	0.67	*V82A*	1.45	0.80	0.34
I76A	4.29	4.16	0.29	*T55S*	0.02	0.30	0.36	*V82G*	3.50	2.83	0.53
I76V	-0.19	0.62	0.17	*T55V*	0.76	1.19	0.28	*V82T*	1.15	1.99	0.53

n	MAE (kcal mol^{-1})	Accuracy (%)	MCC	R^2	ρ	τ
42	0.77	78.6	0.10	0.64	0.79	0.60

Supplementary Table 6. Experimental and calculated changes in free energy for Protein L, B1 domain.

mutation	$\Delta\Delta G_{exp}$	$\Delta\Delta G_{calc}$ (H) ZXZ	SEM	mutation	$\Delta\Delta G_{exp}$	$\Delta\Delta G_{calc}$ (H) ZXZ	SEM	mutation	$\Delta\Delta G_{exp}$	$\Delta\Delta G_{calc}$ (H) ZXZ	SEM
A29G	2.54	3.32	0.19	I6V	0.56	0.73	0.12	T19A	1.11	1.84	0.50
A33G	3.10	3.46	0.11	L10A	3.12	4.27	0.41	T25A	1.25	0.61	0.18
A35G	1.32	1.28	0.24	L40A	2.44	1.15	0.26	T30A	1.09	0.13	0.31
A37G	3.12	3.79	0.11	L58A	3.77	6.28	0.34	T39G	0.17	-0.10	0.31
A52G	0.49	1.00	0.09	F12A	3.12	5.37	0.47	T48A	0.97	0.65	0.23
A8G	2.43	1.51	0.19	F12L	0.68	1.83	0.36	T57A	1.83	0.43	0.22
N14A	1.78	3.74	0.58	F22A	4.25	6.84	0.36	T5A	1.63	1.25	0.34
N44A	0.34	2.17	0.49	F22L	3.12	2.55	0.62	T34G	2.82	5.29	0.22
N59A	1.73	4.32	0.17	F26G	3.08	3.98	0.29	Y36A	2.46	2.95	0.22
N9A	1.87	2.48	0.46	F26L	0.38	0.49	0.25	Y56A	1.66	2.52	0.45
I11A	1.37	3.22	0.20	F62L	3.34	4.07	0.38	Y56L	-0.43	0.17	0.33
I11V	0.47	1.77	0.14	S16A	0.30	-0.09	0.28	V49A	0.92	0.82	0.17
I60A	4.72	7.22	0.26	S31A	-0.41	-0.14	0.56	V4A	1.22	1.76	0.30
I60V	1.69	2.68	0.15	S31G	0.82	0.75	0.61	V51A	1.14	-0.25	0.13
I6A	4.90	5.98	0.21	T17A	1.17	1.24	0.24				

n	MAE (kcal mol^{-1})	Accuracy (%)	MCC	R^2	ρ	τ
44	0.99	90.9	0.31	0.74	0.82	0.64

Supplementary Table 7. Experimental and calculated changes in free energy for c-SRC tyrosine kinase.

mutation	$\Delta\Delta G_{exp}$	$\Delta\Delta G_{calc}$ (H) ZXZ	SEM	mutation	$\Delta\Delta G_{exp}$	$\Delta\Delta G_{calc}$ (H) ZXZ	SEM	mutation	$\Delta\Delta G_{exp}$	$\Delta\Delta G_{calc}$ (H) ZXZ	SEM
A121G	0.92	1.63	0.22	L120A	1.64	2.93	0.29	T96A	-0.06	4.88	0.29
A138G	0.53	0.17	0.20	L124A	0.61	1.36	0.57	T98A	0.01	1.01	0.26
A88G	1.00	1.91	0.17	L89A	1.49	3.67	0.28	W118A	1.29	11.94	0.50
N112A	0.20	1.25	0.64	F102A	0.84	4.51	0.47	W119A	1.20	6.81	0.54
N113A	-0.07	-0.25	0.77	F86A	1.97	3.97	0.61	Y131A	1.52	4.24	0.60
N135A	0.14	0.95	0.48	S101A	0.82	2.64	0.91	Y136A	-0.23	5.99	0.49
Q109A	0.21	1.51	0.52	S123A	1.46	1.77	0.65	Y90A	0.31	1.79	0.44
Q128G	0.35	1.82	0.41	S124A	-0.18	0.14	0.68	Y92A	2.27	13.47	0.59
H122A	0.62	0.61	0.22	S134A	-0.24	-0.57	0.33	Y92F	0.18	4.24	0.28
I110A	0.32	3.60	0.39	S140A	-0.44	-0.83	0.35	V111A	0.77	0.69	0.26
I110V	0.09	0.28	0.23	S94A	-0.52	0.88	0.67	V137A	1.18	1.91	0.40
I132A	1.84	1.47	0.30	T126A	1.79	0.87	0.29	V87A	1.64	2.61	0.30
L100A	1.79	5.41	0.39	T129A	1.11	0.23	0.19				
L108A	2.26	8.02	0.31	T85A	0.64	2.02	0.61				

n	MAE (kcal mol^{-1})	Accuracy (%)	MCC	R^2	ρ	τ
40	2.18	90.0	0.62	0.26	0.52	0.40

Supplementary Table 8. Experimental and calculated changes in free energy for Human lysozyme.

mutation	$\Delta\Delta G_{exp}$	$\Delta\Delta G_{calc}$ (H) ZXZ	SEM	mutation	$\Delta\Delta G_{exp}$	$\Delta\Delta G_{calc}$ (H) ZXZ	SEM	mutation	$\Delta\Delta G_{exp}$	$\Delta\Delta G_{calc}$ (H) ZXZ	SEM
I106A	0.93	2.67	0.40	I59V	1.10	2.91	0.15	T70V	0.69	6.25	0.40
I106V	0.72	2.43	0.18	I89A	2.70	5.18	0.33	Y124F	0.36	1.27	0.48
I23A	2.54	2.64	0.26	I89V	0.48	1.46	0.27	Y20F	0.50	0.72	0.28
I23V	0.36	1.25	0.16	S24A	0.53	-0.91	0.46	Y38F	0.19	1.28	0.34
I56A	3.71	7.09	0.27	S36A	1.12	-2.30	0.69	Y45F	-0.07	0.56	0.19
I56L	0.10	1.85	0.23	S51A	0.24	6.75	0.27	Y54F	0.96	1.59	0.23
I56M	1.77	2.94	0.31	S61A	1.36	3.42	0.43	Y63F	0.24	-0.15	0.20
I56T	3.64	6.23	0.27	S80A	-0.48	0.61	0.62	V100A	0.26	1.21	0.30
I56V	1.20	2.86	0.24	T11A	-0.38	-0.35	0.22	V110A	-0.53	-0.71	0.28
I59A	1.72	6.65	0.20	T11V	-0.31	0.44	0.42	V121A	1.44	0.51	0.34
I59G	3.83	8.35	0.55	T40A	1.51	-0.73	0.26	V125A	1.32	1.92	0.43
I59L	0.00	-1.20	0.23	T40V	1.34	-1.01	0.40	V2A	1.51	1.45	0.16
I59M	1.29	0.63	0.43	T43A	0.36	-0.49	0.19	V74A	0.36	0.24	0.20
I59S	3.59	8.05	0.61	T43V	-0.96	-0.10	0.30	V93A	0.74	-1.38	0.23
I59T	2.22	-1.29	0.46	T52A	0.91	3.93	0.37	V99A	0.98	-1.61	0.51

n	MAE (kcal mol^{-1})	Accuracy (%)	MCC	R^2	ρ	τ
45	1.80	73.3	0.27	0.38	0.43	0.32

Supplementary Table 9. Experimental and calculated changes in free energy for Fibronectin III domain.

mutation	$\Delta\Delta G_{exp}$	$\Delta\Delta G_{calc}$ (H) ZXZ	SEM	mutation	$\Delta\Delta G_{exp}$	$\Delta\Delta G_{calc}$ (H) ZXZ	SEM	mutation	$\Delta\Delta G_{exp}$	$\Delta\Delta G_{calc}$ (H) ZXZ	SEM
A819G	2.80	-0.87	2.89	I849A	2.19	-0.77	1.59	T867A	1.96	-0.77	1.70
A885G	1.09	-0.86	1.55	I850A	2.06	-0.17	2.16	T867S	1.22	-0.46	2.63
I809A	2.86	-0.76	4.38	I8560V	0.83	-0.75	0.83	Y837A	4.22	-0.27	5.30
I809V	0.18	-0.79	0.04	L835A	4.29	-0.56	6.47	Y837F	0.44	-0.72	0.79
I821A	3.67	-0.42	3.95	L851A	2.99	-0.65	4.27	Y858A	1.38	-0.43	2.56
I821V	0.23	-0.72	0.80	L863A	4.22	-0.43	5.34	Y858G	4.14	0.07	5.11
I830A	1.38	-0.17	2.93	L873A	3.06	-0.55	5.54	Y869F	3.13	-0.56	4.26
I830V	0.32	-0.82	1.76	F889A	5.43	-0.67	7.55	V811A	1.36	-0.56	0.99
I833A	3.28	-0.45	5.04	S875A	-0.04	-0.34	1.76	V871A	2.81	-0.82	2.74
I833V	0.50	-0.70	0.86	S882A	2.82	-0.77	1.40				

n	MAE (kcal mol^{-1})	Accuracy (%)	MCC	R^2	ρ	τ
29	0.97	100	1.00	0.82	0.91	0.76

Supplementary Table 10. Experimental and calculated changes in free energy for Trypsin inhibitor.

mutation	$\Delta\Delta G_{exp}$	$\Delta\Delta G_{calc}$ (H) ZXZ	SEM	mutation	$\Delta\Delta G_{exp}$	$\Delta\Delta G_{calc}$ (H) ZXZ	SEM	mutation	$\Delta\Delta G_{exp}$	$\Delta\Delta G_{calc}$ (H) ZXZ	SEM
N24A	2.20	3.70	0.43	L6A	0.60	1.63	0.35	T32A	0.10	0.30	0.21
N44A	3.30	3.53	0.70	M52A	1.70	1.37	0.17	T54A	0.10	0.65	0.46
Q31A	1.00	1.83	0.28	F22A	2.00	0.37	0.46	Y10A	1.20	3.33	0.34
I18A	1.50	2.87	0.26	F4A	3.00	1.76	0.46	Y35A	1.10	7.34	0.44
I19A	2.10	2.65	0.18	S47A	1.60	0.39	0.37	V34A	1.20	1.27	0.22
L29A	0.00	0.07	0.24	T11A	0.00	2.54	0.35				

n	MAE (kcal mol^{-1})	Accuracy (%)	MCC	R^2	ρ	τ
17	1.28	88.2	n.a.	0.07	0.39	0.28

Supplementary Table 11. Experimental and calculated changes in free energy for FK506 binding protein.

mutation	$\Delta\Delta G_{exp}$	$\Delta\Delta G_{calc}$ (H) ZXZ	SEM	mutation	$\Delta\Delta G_{exp}$	$\Delta\Delta G_{calc}$ (H) ZXZ	SEM	mutation	$\Delta\Delta G_{exp}$	$\Delta\Delta G_{calc}$ (H) ZXZ	SEM
I56A	2.48	4.26	0.47	L97A	3.56	4.58	0.40	T75V	0.81	0.25	0.47
I56T	1.81	1.28	0.84	F36A	3.54	2.66	0.66	V101A	2.75	4.92	0.13
I76A	3.81	5.27	0.23	T21A	1.60	1.49	0.50	V23A	2.97	3.60	0.19
I76V	0.76	0.15	0.22	T21S	1.44	1.14	0.42	V24A	3.19	2.63	0.30
I7V	0.94	1.10	0.16	T21V	-0.86	-0.35	0.24	V2A	2.43	2.17	0.17
I91A	1.54	0.70	0.36	T27A	1.97	1.48	0.54	V4A	2.78	3.41	0.22
I91V	0.38	0.12	0.16	T27S	1.49	-0.03	0.35	V55A	2.13	0.79	0.22
I91V	2.32	2.37	0.23	T27V	-0.23	2.01	0.57	V63A	2.97	5.22	0.18
L50A	2.57	6.02	0.37	T75A	2.60	2.01	0.43	V98A	2.16	2.12	0.40

n	MAE (kcal mol^{-1})	Accuracy (%)	MCC	R^2	ρ	τ
27	0.93	92.6	0.46	0.55	0.85	0.68

Supplementary Table 12. Experimental and calculated changes in free energy for Gβ1 domain

mutation	$\Delta\Delta G_{exp}$	$\Delta\Delta G_{calc}$ (H) ZXZ	SEM	*mutation*	$\Delta\Delta G_{exp}$	$\Delta\Delta G_{calc}$ (H) ZXZ	SEM	*mutation*	$\Delta\Delta G_{exp}$	$\Delta\Delta G_{calc}$ (H) ZXZ	SEM
T02A	0.47	1.84	0.47	*T18A*	-0.22	0.56	0.73	*N35A*	-0.59	-0.08	0.53
T02N	0.14	-1.88	0.51	*T18N*	0.87	2.63	0.88	*N35Q*	-0.84	0.32	0.61
T02Q	-0.34	0.56	0.39	*T18Q*	0.59	0.98	1.07	*N35G*	-0.11	-0.18	0.55
T02G	0.67	3.29	0.86	*T18G*	1.74	1.53	0.56	*N35H*	-0.04	0.06	0.83
T02H	0.13	1.21	0.63	*T18H*	0.42	1.26	0.81	*N35I*	-0.68	0.41	0.30
T02I	-1.00	-0.73	0.34	*T18I*	-0.84	1.02	0.61	*N35L*	-0.85	-0.02	0.79
T02L	-0.54	-0.84	0.35	*T18L*	-0.80	-3.02	0.83	*N35M*	-0.18	0.66	0.89
T02M	-1.00	-1.21	0.38	*T18M*	-0.62	2.03	0.85	*N35F*	-1.00	0.06	0.64
T02F	-0.30	1.48	0.41	*T18F*	-0.56	-1.08	0.89	*N35S*	-0.76	0.71	0.64
T02S	-0.06	0.21	0.23	*T18S*	0.78	0.39	0.75	*N35T*	-0.48	-0.53	0.60
T02Y	-0.15	1.67	0.78	*T18Y*	-0.16	-1.18	0.84	*N35Y*	-0.93	0.66	0.84
T02V	-1.31	-0.40	0.22	*T18V*	-0.39	-0.63	0.72	*N35V*	-0.42	1.87	0.53
Y03A	4.00	7.45	0.49	*A20N*	2.85	3.51	0.45	*N37A*	-0.36	0.82	0.42
Y03N	4.00	7.65	0.46	*A20Q*	-0.12	2.77	0.84	*N37Q*	-0.94	0.97	0.68
Y03Q	4.00	6.68	0.36	*A20G*	1.11	1.49	0.15	*N37G*	0.94	1.57	0.32
Y03G	1.44	9.78	0.51	*A20H*	1.91	2.83	0.58	*N37H*	-0.70	0.41	0.35
Y03H	4.00	2.61	0.16	*A20I*	1.86	1.88	0.24	*N37I*	-0.57	-1.85	0.75
Y03I	3.29	4.56	0.30	*A20L*	2.03	3.70	0.35	*N37L*	-0.95	-0.06	0.13
Y03L	3.31	4.37	0.48	*A20M*	1.43	0.93	0.50	*N37M*	-0.30	-0.11	0.38
Y03M	2.60	4.18	0.35	*A20F*	1.73	2.13	0.55	*N37F*	-1.01	1.48	0.57
Y03F	-0.55	0.91	0.19	*A20S*	0.20	0.12	0.18	*N37S*	-0.22	3.58	0.45
Y03S	4.00	6.92	0.66	*A20T*	0.95	0.74	0.28	*N37T*	-0.08	-2.48	0.76
Y03T	4.00	6.89	0.62	*A20Y*	1.94	1.54	1.21	*N37Y*	-0.78	3.09	0.40
Y03V	4.00	5.04	0.42	*A20V*	0.88	1.83	0.24	*N37V*	-0.48	-0.32	0.35
L05A	2.01	4.47	0.28	*V21A*	-0.42	1.15	0.29	*G38A*	0.29	-0.19	0.17
L05N	4.00	-3.05	0.64	*V21N*	-0.50	-0.20	0.37	*G38N*	-0.30	-0.08	0.75
L05Q	4.00	6.36	0.49	*V21Q*	-0.27	0.86	0.58	*G38Q*	-0.19	0.85	0.45
L05G	4.00	4.66	0.36	*V21G*	-0.19	0.97	0.45	*G38H*	-0.50	-0.84	0.31
L05H	4.00	4.61	0.51	*V21H*	-0.19	0.73	0.41	*G38I*	1.44	1.68	0.49
L05I	1.34	2.45	0.30	*V21I*	-0.54	1.20	0.45	*G38L*	-0.04	-0.43	0.35
L05M	0.68	2.92	0.37	*V21L*	-0.41	1.27	0.55	*G38M*	0.47	-0.63	0.24
L05F	0.47	0.47	0.34	*V21M*	-0.52	0.99	0.53	*G38F*	0.20	-0.81	0.53
L05S	4.00	4.67	0.69	*V21F*	-1.10	2.48	0.44	*G38S*	0.17	-0.39	0.82
L05T	4.00	5.72	0.55	*V21S*	-0.44	1.40	0.59	*G38T*	1.01	1.42	0.42
L05Y	2.76	3.95	0.44	*V21T*	-0.13	0.81	0.54	*G38Y*	0.43	-0.06	0.44
L05V	1.25	2.69	0.34	*V21Y*	-0.64	1.40	0.54	*G38V*	1.31	0.77	0.57
I06A	1.25	2.49	0.23	*A23N*	0.19	1.20	0.27	*V39A*	1.18	3.14	0.55
I06N	0.80	-0.55	0.50	*A23Q*	-0.80	0.25	0.38	*V39N*	4.00	3.71	0.36
I06Q	0.58	0.81	0.35	*A23G*	0.83	1.50	0.19	*V39Q*	1.52	2.75	0.53
I06G	3.08	3.68	0.26	*A23H*	0.20	0.43	0.29	*V39G*	2.29	3.59	0.57
I06H	1.06	1.57	0.33	*A23I*	-0.56	1.78	0.29	*V39H*	1.57	1.88	0.59
I06L	0.11	0.34	0.31	*A23L*	-0.22	1.35	0.26	*V39I*	-0.91	0.27	0.21
I06M	0.50	0.71	0.17	*A23M*	-0.51	0.69	0.31	*V39L*	-0.27	-0.22	0.44
I06F	0.10	1.57	0.34	*A23F*	-0.59	1.18	0.26	*V39M*	0.27	1.62	0.65
I06S	1.38	2.58	0.30	*A23S*	-0.16	1.33	0.35	*V39F*	-0.67	-1.11	0.38
I06T	0.91	-0.74	0.76	*A23T*	-0.32	1.55	0.23	*V39S*	1.70	3.58	0.34
I06Y	0.21	1.71	0.30	*A23Y*	-0.38	1.11	0.30	*V39T*	1.28	0.18	0.84
I06V	-0.49	0.54	0.15	*A23V*	-0.38	0.91	0.20	*V39Y*	0.42	0.91	0.59
L07A	1.40	5.12	0.51	*A24N*	0.24	-0.54	0.14	*G41A*	-0.49	1.56	0.28
L07N	4.00	4.71	0.26	*A24Q*	-0.05	0.65	0.33	*G41N*	4.00	-	-
L07Q	1.54	2.84	0.37	*A24G*	-0.15	-0.06	0.13	*G41Q*	4.00	1.11	0.64
L07G	4.00	7.29	0.36	*A24H*	0.39	-1.18	0.30	*G41H*	4.00	-	-
L07H	1.59	4.05	0.51	*A24I*	-0.55	0.21	0.16	*G41I*	-0.58	4.93	0.79
L07I	-0.97	0.69	0.14	*A24L*	-0.27	0.74	0.20	*G41L*	4.00	0.81	0.58
L07M	-0.50	0.57	0.33	*A24M*	-0.41	0.58	0.32	*G41M*	-0.19	-0.27	0.43
L07F	-0.26	0.88	0.33	*A24F*	-0.62	-0.13	0.27	*G41F*	2.32	0.73	0.63
L07S	1.79	4.55	0.52	*A24S*	-0.22	-0.51	0.20	*G41S*	4.00	3.91	0.55
L07T	1.14	4.18	0.51	*A24T*	-0.23	0.96	0.72	*G41T*	4.00	1.53	1.05
L07Y	0.02	2.05	0.37	*A24Y*	-0.44	0.21	0.32	*G41Y*	2.49	0.90	0.84
L07V	-0.08	1.28	0.27	*A24V*	-0.19	0.09	0.18	*G41V*	4.00	-	-
N08A	0.28	2.30	0.93	*T25A*	-0.85	2.15	0.34	*T44A*	-0.40	-0.16	0.32
N08Q	-0.29	2.66	0.89	*T25N*	-0.02	2.67	0.48	*T44N*	0.00	0.21	0.44
N08G	0.85	2.30	0.89	*T25Q*	-0.47	2.96	0.47	*T44Q*	-0.22	-0.08	0.54
N08H	-0.24	2.22	0.41	*T25G*	-0.17	2.55	0.50	*T44G*	0.18	-0.39	0.44
N08I	-0.56	1.27	0.54	*T25H*	-0.67	3.17	0.47	*T44H*	-0.31	0.13	0.59
N08L	-0.95	-0.53	0.65	*T25I*	0.35	4.02	0.74	*T44I*	-0.52	1.05	0.42
N08M	-0.37	0.75	0.82	*T25L*	0.06	3.98	0.26	*T44L*	-0.07	1.42	0.24
N08F	-0.35	0.74	0.29	*T25M*	-0.20	2.12	0.33	*T44M*	0.11	1.09	0.59
N08S	0.04	1.98	0.76	*T25F*	-0.39	4.70	0.50	*T44F*	-0.09	0.14	0.37
N08T	0.61	2.27	1.10	*T25S*	-0.51	2.10	0.58	*T44S*	-0.36	-1.24	0.47

N08Y	-0.21	0.48	0.43
N08V	-0.13	2.25	0.53
G09A	0.25	-0.32	0.30
G09N	1.29	0.61	1.15
G09Q	1.06	1.48	0.58
G09H	1.22	0.86	0.70
G09I	0.59	-1.01	0.55
G09L	-0.32	0.75	0.61
G09M	0.55	0.60	0.40
G09F	0.00	4.77	0.91
G09S	-0.03	1.12	0.70
G09T	-0.30	1.19	0.61
G09Y	0.54	2.02	1.02
G09V	0.42	0.61	0.59
T11A	-0.24	-0.38	0.86
T11N	-0.17	-1.30	0.50
T11Q	-0.59	-0.89	0.29
T11G	0.01	0.15	0.60
T11H	-0.42	-0.41	0.69
T11I	-0.56	-0.10	0.72
T11L	-0.38	0.52	0.53
T11M	-0.39	0.67	0.54
T11F	-0.76	-0.16	0.67
T11S	-0.30	0.54	0.17
T11Y	-0.30	-0.79	0.54
T11V	-0.54	0.53	0.54
L12A	0.28	1.23	0.48
L12N	-0.09	0.41	0.65
L12Q	-0.14	1.70	0.56
L12G	0.28	1.24	0.32
L12H	-0.28	-0.28	0.44
L12I	-0.99	-0.02	0.36
L12M	-0.34	-0.30	0.23
L12F	-1.26	-1.13	0.33
L12S	0.06	1.13	0.38
L12T	-0.42	0.73	0.85
L12Y	-1.78	-1.35	0.28
L12V	-0.63	-0.14	0.36
G14A	-0.51	0.17	0.21
G14N	-0.03	-1.23	0.91
G14Q	-0.52	-1.06	0.55
G14H	-0.08	0.47	0.57
G14I	-0.81	-0.68	0.30
G14L	-1.25	-0.29	0.48
G14M	-0.94	0.04	0.40
G14F	-0.67	-0.66	0.52
G14S	-0.51	0.65	0.51
G14T	-0.39	0.24	0.69
G14Y	-0.24	0.21	0.93
G14V	-0.52	0.47	0.39
T16A	-0.26	-1.54	0.46
T16N	0.14	0.81	0.34
T16Q	-0.33	1.88	0.71
T16G	1.21	-0.57	0.40
T16H	-0.51	0.04	0.47
T16I	-1.01	-3.52	0.43
T16L	-0.97	-1.30	0.37
T16M	-0.70	-1.45	0.28
T16F	-1.85	-2.06	0.57
T16S	-0.01	-2.49	0.36
T16Y	-1.31	-1.01	0.58
T16V	-0.50	-2.82	0.28
T17A	0.21	-0.16	0.23
T17N	0.13	-0.78	0.26
T17Q	-0.28	-0.85	0.38
T17G	1.05	1.51	0.33
T17H	0.05	-1.79	0.51
T17I	-0.06	-1.49	0.23
T17L	0.07	-2.11	0.47
T17M	-0.06	-0.75	0.24
T17F	-0.68	-0.30	0.68
T17S	-0.39	0.20	0.30
T17Y	-0.71	-2.62	0.87
T17V	-0.18	-1.31	0.46
V54A	1.89	2.25	0.31

T25Y	-0.10	4.22	0.29
T25V	0.10	3.56	0.22
A26N	4.00	8.56	0.65
A26Q	4.00	2.70	0.94
A26G	4.00	3.95	0.12
A26H	4.00	11.05	0.59
A26I	4.00	4.79	0.62
A26L	4.00	6.22	0.71
A26M	4.00	3.94	0.57
A26F	4.00	10.20	0.90
A26S	2.33	3.82	0.17
A26T	4.00	5.67	0.49
A26Y	4.00	11.47	0.83
A26V	2.41	2.76	0.56
V29A	-0.14	0.18	0.21
V29N	0.22	-0.17	0.20
V29Q	-0.38	0.18	0.36
V29G	0.91	2.02	0.28
V29H	-0.16	-0.65	0.28
V29I	-0.68	-0.69	0.19
V29L	-0.29	-1.24	0.23
V29M	-0.14	0.00	0.21
V29F	-0.59	-0.61	0.28
V29S	0.17	0.30	0.16
V29T	-0.41	0.21	0.31
V29Y	-0.19	0.07	0.23
F30A	3.04	2.43	0.47
F30N	4.00	4.78	0.47
F30Q	4.00	4.88	0.32
F30G	4.00	5.12	0.45
F30H	4.00	2.72	0.53
F30I	4.00	2.78	0.27
F30L	0.48	1.13	0.32
F30M	1.66	1.13	0.30
F30S	4.00	4.89	0.80
F30T	4.00	1.19	0.74
F30Y	-0.91	4.51	0.21
F30V	2.53	1.22	0.45
Q32A	-0.16	0.03	0.43
Q32N	0.01	-0.85	1.40
Q32G	0.42	0.73	0.41
Q32H	0.20	0.81	0.35
Q32I	-0.11	0.69	0.25
Q32L	-0.14	1.09	0.21
Q32M	-0.50	1.13	0.23
Q32F	0.32	1.62	0.26
Q32S	-0.20	-0.51	0.54
Q32T	-0.32	0.18	0.94
Q32Y	0.39	1.73	0.26
Q32V	0.13	1.32	0.23
Y33A	1.77	2.65	0.34
Y33N	1.95	-0.86	1.02
Y33Q	1.34	1.23	0.31
Y33G	3.46	3.72	0.45
Y33H	1.44	0.28	0.36
Y33I	0.14	1.35	0.42
Y33L	0.72	-0.16	0.34
Y33M	0.60	0.88	0.34
Y33F	-0.29	-0.39	0.30
Y33S	2.09	2.30	0.46
Y33T	1.64	-2.98	0.39
Y33V	0.44	1.51	0.34
A34N	1.46	3.77	0.26
A34Q	2.33	4.29	0.64
A34G	1.46	2.09	0.28
A34H	3.19	3.54	0.66
A34I	0.67	1.06	0.34
A34L	-0.11	0.58	0.41
A34M	0.45	1.65	0.87
A34F	1.85	1.30	0.69
A34S	0.53	2.64	0.24
A34T	0.38	2.32	0.85
A34Y	2.00	3.29	0.45
A34V	-0.02	1.18	0.25
V54S	1.54	2.81	0.32

T44Y	-0.40	1.01	0.37
T44V	0.10	0.87	0.55
Y45A	4.00	7.98	0.21
Y45N	4.00	7.17	0.77
Y45Q	4.00	9.32	0.46
Y45G	4.00	9.00	0.27
Y45H	4.00	5.67	0.15
Y45I	2.24	6.60	0.26
Y45L	1.79	6.13	0.17
Y45M	2.49	4.70	0.40
Y45F	0.05	1.89	0.18
Y45S	2.97	8.14	0.38
Y45T	4.00	8.58	0.56
Y45V	2.53	6.75	0.21
A48N	-0.13	-0.96	0.27
A48Q	-0.54	-0.22	0.15
A48G	0.12	-0.85	0.08
A48H	0.08	-0.15	0.30
A48I	-0.07	0.21	0.25
A48L	-0.51	0.44	0.20
A48M	0.04	0.13	0.19
A48F	0.00	-0.22	0.21
A48S	-0.29	-0.53	0.23
A48T	-0.16	1.31	0.38
A48Y	-0.41	0.02	0.26
A48V	-0.33	0.01	0.19
T49A	0.56	2.36	0.24
T49N	-0.07	2.62	0.66
T49Q	0.39	2.42	0.26
T49G	0.91	1.82	0.43
T49H	0.47	1.44	0.42
T49I	0.33	2.38	0.34
T49L	-0.21	3.02	0.28
T49M	0.45	3.50	0.17
T49F	0.69	3.73	0.23
T49S	-0.18	2.77	0.66
T49Y	0.83	3.63	0.23
T49V	0.50	1.28	0.34
T51A	1.10	2.37	0.47
T51N	2.19	2.78	0.49
T51Q	1.03	1.64	0.57
T51G	2.48	3.82	0.43
T51H	2.16	2.02	0.81
T51I	0.30	1.42	0.22
T51L	1.05	1.26	0.20
T51M	0.98	3.71	0.45
T51F	4.00	4.04	0.82
T51S	0.62	4.41	0.59
T51Y	1.64	0.72	0.28
T51V	0.50	1.14	0.25
F52A	4.00	7.33	0.53
F52N	4.00	6.02	0.49
F52Q	4.00	6.52	0.48
F52G	4.00	11.77	0.37
F52H	2.66	5.08	0.19
F52I	0.24	5.05	0.27
F52L	2.53	5.05	0.18
F52M	2.71	2.63	0.43
F52S	4.00	8.63	0.50
F52T	4.00	9.59	0.54
F52Y	0.34	1.94	0.29
F52V	2.99	5.49	0.29
T53A	1.18	-1.13	0.34
T53N	0.65	0.29	0.47
T53Q	0.35	-2.49	0.58
T53G	4.00	0.98	0.54
T53H	0.98	-2.88	0.68
T53I	0.05	-3.01	0.65
T53L	0.37	-3.01	0.59
T53M	-0.15	-3.03	0.56
T53F	0.02	-3.70	0.72
T53S	0.44	-1.45	0.51
T53Y	-0.24	-3.45	0.93
T53V	0.11	-1.44	0.48
T55G	0.98	1.73	0.78

V54N	4.00	-1.22	0.40	*V54T*	1.68	2.54	0.26	*T55H*	0.66	0.87	0.68
V54Q	2.91	3.35	0.38	*V54Y*	1.73	3.65	0.62	*T55I*	0.24	0.40	0.48
V54G	4.00	4.29	0.31					*T55L*	0.30	-0.08	0.26
V54H	4.00	3.82	0.49					*T55M*	0.48	0.53	0.25
V54I	-0.26	0.93	0.27					*T55F*	0.19	0.07	0.39
V54L	0.08	-0.74	0.25	*T55A*	0.70	0.98	0.34	*T55S*	-0.35	0.79	0.49
V54M	0.68	1.05	0.51	*T55N*	-0.34	1.65	0.98	*T55Y*	0.14	1.05	0.59
V54F	1.64	1.00	0.60	*T55Q*	0.04	0.44	0.33	*T55V*	0.23	0.78	0.67

n	MAE (kcal mol^{-1})	Accuracy (%)	MCC	R^2	ρ	τ	
399[a] (456[b])	1.27	60.0 (64.5)	0.22 (0.27)	0.30	0.48	0.34	

[a] Includes only experimental quantitative values.

[b] Includes also (n = 57) qualitative (capped) experimental values. For these datapoints, only qualitative statistical figures of merit (i.e., accuracy and MCC) are meaningful.

Supplementary Table 13. Analysis of the wild-type and mutant residue types for Gβ1 domain.

From	MAE (kcal mol^{-1})	*n*	To	MAE (kcal mol^{-1})	*n*
ALA	0.99	72	ALA	1.11	32
ASN	1.46	36	ASN	1.04	35
GLN	0.81	12	GLN	1.46	37
GLY	1.47	48	GLY	1.15	34
HIS	-	-	HIS	1.15	38
ILE	0.93	12	ILE	1.53	37
LEU	1.35	36	LEU	1.46	35
MET	-	-	MET	1.08	38
PHE	1.13	24	PHE	1.51	36
SER	-	-	SER	1.58	38
THR	1.42	132	THR	1.56	27
TYR	1.41	36	TYR	1.61	35
VAL	1.14	48	VAL	1.16	34

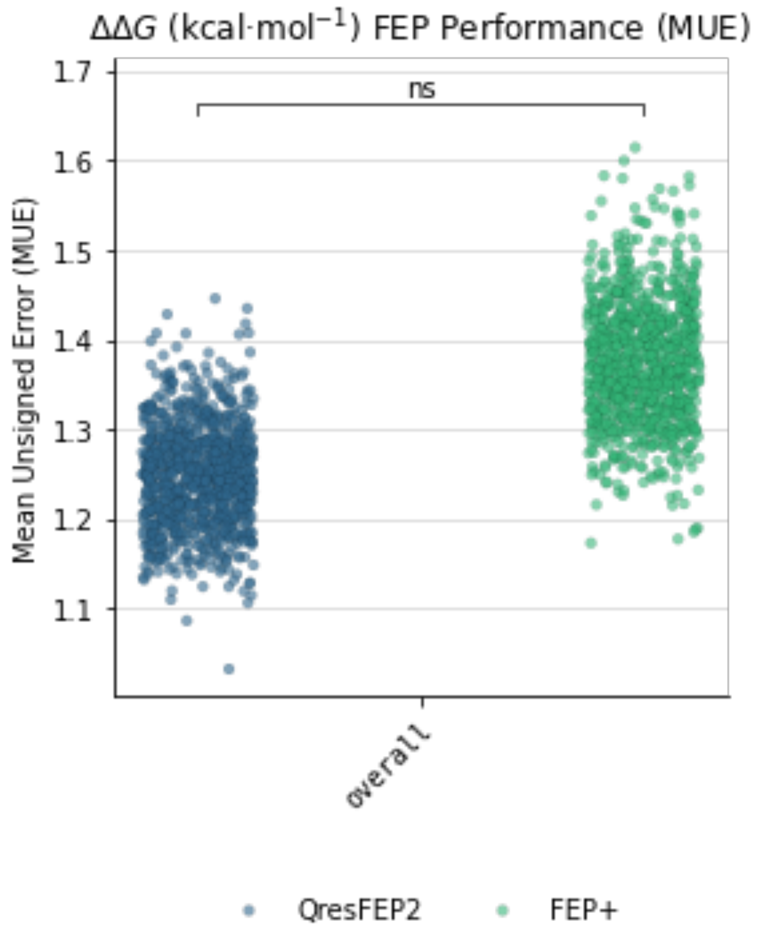

Supplementary Figure 15. Statistical comparison of the accuracy based on the mean unassigned error (MUE, kcal·mol^{-1}) of QresFEP-2 (blue dots) and FEP+ (green dots), calculated for the common 534 datapoints of the 10 protein systems comprising the benchmark dataset (Table 4). Individual MUE values obtained by 1,000 cycles of bootstrapping. **** Indicates statistically significant lower MUE for QresFEP-2 based on the Mann-Whitney-Wilcoxon non-parametric test ($p < 0.0001$)

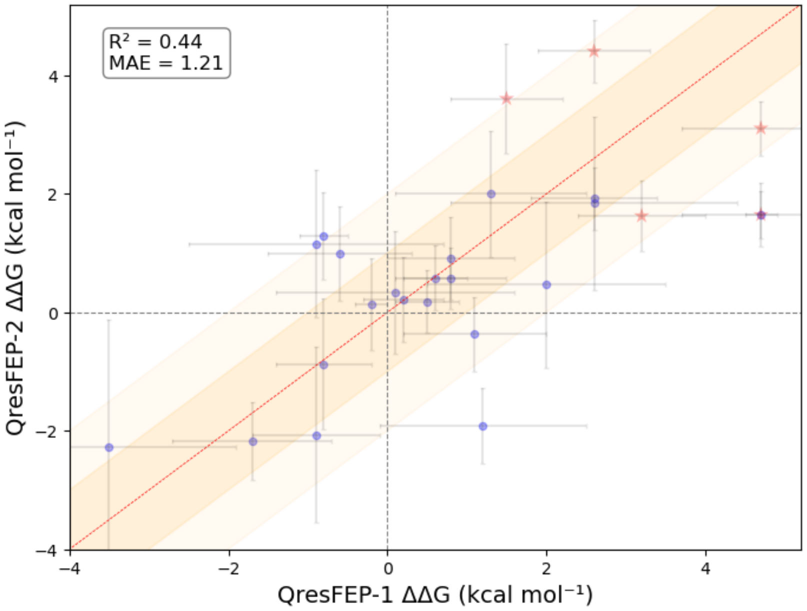

Supplementary Figure 16. Autocorrelation plots for A_{2A}-NECA site-directed mutagenesis mutations between the new hybrid-topology QresFEP-2 protocol and single-topology stepwise annihilation QresFEP-1 protocol. Error bars correspond to the SEM values obtained with each method. Points denoted with a star (⋆) correspond to mutations with no detectable binding above the concentration used in experimental assays.

Supplementary Table 14. Experimental and calculated changes in free energy for Adenosine A_{2A} receptor in complex with NECA.

mutation	$\Delta\Delta G_{exp}$	$\Delta\Delta G_{calc}$ (H) ZXZ	SEM	mutation	$\Delta\Delta G_{exp}$	$\Delta\Delta G_{calc}$ (H) ZXZ	SEM	mutation	$\Delta\Delta G_{exp}$	$\Delta\Delta G_{calc}$ (H) ZXZ	SEM
N181S	-0.10	-0.87	1.10	*S277T*	0.40	2.00	1.07	*M177A*	-0.20	1.92	0.52
Q89D	-1.60	-2.28	2.16	*T88S*	2.20	0.91	0.70	*F168A*	1.40	3.11	0.46
H250N	-0.80	1.16	1.25	*V84L*	0.00	-0.37	0.63	*F180A*	0.50	1.29	0.73
H250F	0.40	-1.91	0.64	*N253A*	2.30	4.41	0.53	*S277A*	3.50	0.18	0.53
H250Y	0.50	0.34	1.03	*C254A*	-0.10	0.22	0.72	*S90A*	-0.90	0.14	0.78
F182W	1.20	0.99	0.79	*Q89A*	-1.60	-2.07	1.48	*S91A*	0.20	0.58	0.55
F182Y	1.30	0.57	0.52	*H278A*	2.30	1.84	1.46	*T88A*	2.60	1.65	0.40
S277N	0.50	0.47	1.40	*H250A*	2.30	3.61	0.92	*V84A*	1.40	1.65	0.53
S277C	0.90	-2.17	0.66	*I274A*	2.30	1.63	0.59				

n	MAE (kcal mol^{-1})	Accuracy (%)	MCC	R^2	ρ	τ	
26	1.12	76.92	0.43	0.31	0.53	0.41	

Supplementary Table 15. Experimental and calculated changes in free energy for the Barnase-Barstar PPI system.

mutation	$\Delta\Delta G_{exp}$	$\Delta\Delta G_{calc}$ (H) ZXZ	SEM	mutation	$\Delta\Delta G_{exp}$	$\Delta\Delta G_{calc}$ (H) ZXZ	SEM	mutation	$\Delta\Delta G_{exp}$	$\Delta\Delta G_{calc}$ (H) ZXZ	SEM
H102A	6.43	8.14	0.44	*N58A*	3.09	1.30	0.50	*T42A*	1.86	1.72	0.48
H102Q	4.55	6.96	1.25	*W35F*	1.26	1.15	0.29	*W38F*	1.64	0.90	0.63
H102G	6.82	12.28	0.70	*Y29A*	3.47	3.02	0.55	*W44F*	0.06	1.57	0.46
H102L	7.66	5.58	0.86	*Y29F*	-0.13	-1.79	0.98				

n	MAE (kcal mol^{-1})	Accuracy (%)	MCC	R^2	ρ	τ	
11	1.64	100	1.00	0.72	0.86	0.71	
