# Supplementary material for: Accurate predictions of protein mutational effects accelerated with a hybrid-topology free energy protocol
Source: Commun Chem. 2025 Nov 20;8:362. doi: 10.1038/s42004-025-01771-0 (PMC12634679; doi:10.1038/s42004-025-01771-0)
Supplement: Supplementary file 3 — Description of Additional Supplementary Files [file 42004_2025_1771_MOESM3_ESM.pdf]

Description of Additional Supplementary Files

File name: Supplementary Data 1

Description: Data described in the results and used to produce all Figures and Tables

File name: Supplementary Movie 1

Description: Trajectories corresponding to the hybrid-topology Q-resFEP2 transformation of Tyr (magenta) to Phe (orange) in position 24 of barnase ribonuclease, using the four different restraining schemes depicted on Fig. 2
